# Type I conventional dendritic cells and CD8^+^ T cells predict favorable clinical outcome of head and neck squamous cell carcinoma patients

**DOI:** 10.3389/fimmu.2024.1414298

**Published:** 2024-06-13

**Authors:** Johanna Kirchner, Ioana Plesca, Rebecca Rothe, Antonia Resag, Steffen Löck, Iva Benešová, Luise Rupp, Annett Linge, Rebekka Wehner, Mechthild Krause, Marc Schmitz

**Affiliations:** ^1^ OncoRay – National Center for Radiation Research in Oncology, Faculty of Medicine and University Hospital Carl Gustav Carus, Technische Universität Dresden and Helmholtz-Zentrum Dresden – Rossendorf (HZDR), Dresden, Germany; ^2^ Department of Radiotherapy and Radiation Oncology, Faculty of Medicine and University Hospital Carl Gustav Carus, Technische Universität Dresden, Dresden, Germany; ^3^ Faculty of Medicine Carl Gustav Carus, Institute of Immunology, Technische Universität Dresden, Dresden, Germany; ^4^ National Center for Tumor Diseases (NCT), Partner Site Dresden; German Cancer Research Center (DKFZ), Heidelberg, Germany; ^5^ German Cancer Consortium (DKTK), partner site Dresden and German Cancer Research Center (DKFZ), Heidelberg, Germany; ^6^ Helmholtz-Zentrum Dresden-Rossendorf (HZDR), Institute of Radiooncology – OncoRay, Dresden, Germany

**Keywords:** dendritic cells, head and neck squamous cell carcinoma, human papilloma virus, hypoxia, multiplex imaging, spatial biology, T cells, tumor microenvironment

## Abstract

Head and neck squamous cell carcinoma (HNSCC) is one of the most common tumor entities worldwide, with human papillomavirus (HPV) infection contributing to cancer development. Conventional therapies achieve only limited efficiency, especially in recurrent or metastatic HNSCC. As the immune landscape decisively impacts the survival of patients and treatment efficacy, this study comprehensively investigated the immunological tumor microenvironment (TME) and its association with patient outcome, with special focus on several dendritic cell (DC) and T lymphocyte subpopulations. Therefore, formalin-fixed paraffin-embedded tumor samples of 56 HNSCC patients, who have undergone resection and adjuvant radiotherapy, were analyzed by multiplex immunohistochemistry focusing on the detailed phenotypic characterization and spatial distribution of DCs, CD8^+^ T cells, and T-helper cell subsets in different tumor compartments. Immune cell densities and proportions were correlated with clinical characteristics of the whole HNSCC cohort and different HPV- or hypoxia-associated subcohorts. Tumor stroma was highly infiltrated by plasmacytoid DCs and T lymphocytes. Among the T-helper cells and CD8^+^ T cells, stromal regulatory T cells and intraepithelial exhausted CD8^+^ T cells expressing programmed cell death protein-1 (PD-1^+^) and/or lymphocyte-activation gene-3 (LAG-3^+^) were the predominant phenotypes, indicating an immunosuppressive TME. HPV-associated tumors showed significantly higher infiltration of type I and type II conventional DCs (cDC1, cDC2) as well as several CD8^+^ T cell phenotypes including exhausted, activated, and proliferating T cells. On the contrary, tumors with hypoxia-associated gene signatures exhibited reduced infiltration for these immune cells. By multivariate Cox regression, immune-related prognostic factors were identified. Patient clusters defined by high infiltration of DCs and T lymphocytes combined with HPV positivity or low hypoxia showed significantly prolonged survival. Thereby, cDC1 and CD8^+^ T cells emerged as independent prognostic factors for local and distant recurrence. These results might contribute to the implementation of an immune cell infiltration score predicting HNSCC patients’ survival and such patient stratification might improve the design of future individualized radiochemo-(immuno)therapies.

## 1 Introduction

With estimated 880,000 new cases and 450,000 deaths worldwide in 2020, head and neck squamous cell carcinoma (HNSCC) is the sixth most common tumor entity and incidences continue to rise, with a growing proportion of human papillomavirus (HPV)-infection driven tumors over the last decades (1). HNSCC includes tumors located in the oral cavity, oropharynx, hypopharynx, and larynx. Besides some well-known risk factors, such as extensive consumption of tobacco and alcohol, the infection with HPV (primarily genotype 16) causes carcinogenesis in roughly 25% of HNSCC, particularly in the oropharynx (> 70%), hence representing a major leading factor for HNSCC development (2).

Despite the advanced treatment regimen consisting of radical resection and adjuvant high-dose radio(chemo)therapy (R(C)Tx) or definite R(C)Tx in locally advanced HNSCC, the 5-year overall survival (OS) rate of 50-69% still can be improved (3). Moreover, limited prognosis also results from frequent local relapse occurring in up to 60% of HNSCC patients (4). The implementation of immunotherapy approaches, such as anti-programmed cell death protein-1 (anti-PD-1) antibodies, was intended to improve survival outcomes. However, this was noticeable only in 15-30% of HNSCC patients with recurrent or metastatic disease (5, 6). Several studies highlighted that HPV-associated (HPV+) oropharyngeal tumors are generally linked to an improved clinical outcome following R(C)Tx, compared to the HPV-negative (HPV-) ones. Hence, these HPV+ tumors constitute a distinct cancer entity (7, 8), with p16 upregulation being used as their corresponding marker in the 8^th^ edition of tumor, node, metastasis (TNM) classification, even though the use of p16 overexpression alone as a prognostic and diagnostic marker needs further validation (9). Other parameters, including the combination of HPV-DNA/RNA and p16, hypoxia and hypoxia-induced gene expression, high expression of cancer stem cell markers, tumor volume, solute carrier family 3 member 2 (SLC3A2) and CD44 protein expression, have also been described as prognostic factors for local relapse after R(C)Tx in HNSCC (10). For instance, hypoxia-associated gene signatures or a hypoxic tumor microenvironment (TME) are associated with reduced therapeutic efficacy and poor prognosis (11, 12).

Despite the utility of tumor localization, p16 status, and hypoxia in subgrouping HNSCC patients and predicting clinical outcome, there is still a high variability in treatment response. Therefore, novel biomarkers as well as improved tools to predict therapeutic success and individualize treatment modalities are urgently needed to optimize therapy efficacy, properly adjust therapeutic regimens, and prevent over- or under-treatment with side effects or treatment failure (13).

HNSCC is known to be an immunogenic tumor entity including a high tumor infiltration of myeloid and lymphoid cells (14). However, tumor cells generate an immunosuppressive microenvironment resulting, for instance, in enhanced infiltration of activated regulatory T cells (T_regs_) and generation of exhausted T cells (5). Thus, the tumor immune contexture comprising the frequency, spatial distribution, orientation, and functional characteristics of tumor-infiltrating immune cells plays a critical role for the clinical outcome of cancer patients (15, 16). A positive correlation between high densities of tumor-infiltrating T cell subsets, in particular CD8^+^ T cells, and improved prognosis has been reported for various cancer entities, including HNSCC (15, 17). Additionally, dendritic cells (DCs) are known as key players of innate and adaptive immunity (18). DCs are clinically relevant, as demonstrated in various cancer entities (19, 20), and may also significantly impact the outcome of HNSCC patients (21). The function of cancer-infiltrating immune cells is dual and context-dependent, as they can either favor anticancer immunity or promote tumor tolerance. Hence, their role has to be described individually for each cancer entity and immune cell subtype (22). For instance, plasmacytoid DCs (pDCs), described as the main producers of type I interferon upon stimulation, can either participate in antitumor immunity and favorably impact clinical outcome (20), or they may adopt a tolerogenic phenotype and thereby contribute to poor prognosis (23). Other studies investigating the tumor immune architecture revealed that infiltrating T cells also significantly influenced the clinical efficacy of various treatments, including R(C)Tx (24, 25).

In HNSCC, the role of the immunological TME is subject of ongoing research. However, there are limited investigations exploring the tumor immune landscape in detail. So far, most studies have focused on detecting the presence of T cells using immunohistochemistry or bulk gene expression profiles of tumor samples (26–29). Several groups reported a positive impact of high densities of CD8^+^ T cells on the survival and relapse of HNSCC patients (5, 27). Nonetheless, these previous studies generally lack a more detailed phenotypic description of infiltrating CD8^+^ T lymphocytes in terms of their phenotype orientation, for instance by assessing the (co-)expression of activating and/or inhibitory markers, their localization relative to the tumor cells, and whether their spatial distribution impacts survival. Moreover, little is known about the infiltration pattern and clinical influence of distinct DC and T-helper (T_H_) subsets in HNSCC.

Here, we aimed to explore these unresolved issues by comprehensively investigating the presence and role of several tumor-infiltrating T cell subpopulations, two conventional DC subtypes (cDC1 and cDC2), as well as pDCs in more detail, characterizing their phenotype and spatial distribution in distinct compartments of HNSCC tissue samples by multiplex immunohistochemistry (mIHC). We correlated the densities and proportions of various immune cell subsets with clinical characteristics of the whole HNSCC cohort and different HPV- or hypoxia-associated subcohorts. In addition, we used survival parameters and clinicopathological characteristics of the patient cohort to identify immune cell types with an independent prognostic value. Thereby, we provide a more comprehensive picture of the HNSCC immune architecture supporting the identification of novel prognostic biomarkers for this cancer entity.

## 2 Materials and methods

### 2.1 Patients and study design

HNSCC patients were enrolled after the Ethics Committee of Technische Universität Dresden approved this retrospective analysis of clinical and biological data (No EK 397102014). Informed consent was given by patients before treatment. Eligible patients (n = 56) had histologically proven squamous cell carcinoma of the oral cavity or oropharynx in an advanced stage. All patients were treated with curatively intended radical R0 resection followed by adjuvant RTx between 09/2005 and 10/2016 with a total dose of 60-66 Gy, covering the tumor region and regional lymph nodes and including a boost to the tumor region and to positive lymph node levels according to standard protocols. Patients with p16+ tumors were included if they were also HPV-DNA or HPV-RNA positive, according to a method evaluated and described before (30). Clinicopathological characteristics of the patient cohort were summarized in **Supplementary Table 1**.

### 2.2 Biomarker analysis and immunohistochemical stainings

To assess clinicopathological parameters of the patient cohort, immunohistochemical staining of p16, DNA extraction and PCR-array based analyses of HPV status, as well as NanoString RNA analyses were conducted similar to other German Cancer Consortium (DKTK) cohorts, as described previously (10). Briefly, p16 overexpression (also called p16 positivity) was defined as ≥ 70% intense tumor staining using the immunohistochemical CINtec Histology kit (Roche mtm laboratories AG, Basel, Switzerland). Negative controls were included to confirm the positive stainings. HPV-DNA analysis was performed with the LCD-Array HPV 3.5 kit (CHIPRON GmbH, Berlin, Germany) following DNA extraction from 5 µm formalin-fixed paraffin-embedded (FFPE) sections with QIAamp DNA FFPE tissue kit (QIAGEN N.V., Venlo, Netherlands). Hypoxia status was determined by gene expression analysis, using the NanoString Elements technology (NanoString Technologies, Seattle, Washington, USA) and the previously established 15-gene hypoxia-associated signature (Hypox15) (31).

### 2.3 Multiplex immunohistochemistry

To detect tumor-infiltrating immune cell populations, mIHC of 3-5 µm FFPE tumor sections was performed using the tyramide signal amplification-based OPAL technology (Akoya Biosciences, Marlborough, Massachusetts, USA) on the Ventana Ultra Instrument (Ventana Medical Systems, Basel, Switzerland) as described before (32, 33). First, tissue sections were deparaffinized and rehydrated for 24 min at 69°C in EZ Prep solution (Ventana Medical Systems). Subsequently, heat-mediated antigen retrieval was performed for 32 min at 95°C in Cell Conditioning solution 1 (Ventana Medical Systems). Tissue sections were incubated with primary antibodies for 32 min (blood dendritic cell antigen-2 [BDCA-2], interferon regulatory factor 7 [IRF7], C-type lectin domain family 9 member A [CLEC9A], C‐type lectin domain family 10 member A [CLEC10A], CD1c, CD3, CD8, granzyme B [GrzB], Ki67, forkhead box P3 [FoxP3], T-box expressed in T cells [T-bet], GATA binding protein 3 [GATA3], pan-cytokeratin [PanCK]) or 60 min (PD-1, LAG-3, RAR-related orphan receptor gamma [RORγT]) at 36°C. Next, anti-species secondary antibodies (OmniMAP HRP anti-rabbit, OmniMAP HRP anti-mouse, DISCOVERY anti-mouse HQ, Ventana Medical Systems) and optionally tertiary antibodies (DISCOVERY anti-HQ HRP) were added to the tissues for 12 min at 36°C. Finally, tissues were incubated with Opal fluorophores (Akoya Biosciences) for 8 min at room temperature. Antibodies were stripped by heating the samples in Cell Conditioning solution 2 (Ventana Medical Systems) for 24 min at 100°C (8 min for PanCK in the DC panel). Antibody incubation was repeated sequentially for all antibodies of the panel. Staining procedure was finalized by counterstaining with DAPI (Merck KGaA, Darmstadt, Germany). Tissues were mounted in Fluoromount-G® medium (SouthernBiotech, Birmingham, Alabama, USA).

In total, three different antibody panels were stained allowing for the detection of five distinct immune cell marker in addition to the tumor marker PanCK and DAPI nuclear stain (**Supplementary Table 2**). To assess the phenotype and frequency of DCs, a panel consisting of CLEC9A (cDC1), CD1c and CLEC10A (both for cDC2), BDCA-2 (pDCs), and the transcription factor (TF) IRF7 (activation marker for pDC, nuclear expression) was stained. To differentiate several functional phenotypes of cytotoxic T cells, the second mIHC panel comprised the lineage marker CD8, the inhibitory immune checkpoints LAG-3 and PD-1, the activation marker GrzB, and the proliferation marker Ki67. Lastly, to analyze the proportion of T_H_ subtypes a panel with the lineage marker CD3 and the TFs T-bet (T_H_1 cells), GATA3 (T_H_2 cells), RORγT (T_H_17 cells), and FoxP3 (T_regs_) was stained. Detailed information about the antibody panels are summarized in **Supplementary Table 2**.

### 2.4 Image analysis and cell quantification

Images were acquired by the Vectra 3.0 Automated Imaging System (Akoya Biosciences). Randomly set multiplex images were taken at 200× magnification in a 25% coverage of the whole tumor area (WTA, **Supplementary Figure 1**). Image data was processed with the inForm® software (Akoya Biosciences) using a semi-automatic approach. Algorithms were trained to differentiate tissue from non-tissue, and epithelial from stromal tissue based on PanCK positivity (**Supplementary Figure 2A**). Cells were segmented based on DAPI staining and phenotyped based on the signal intensity of the respective marker (**Supplementary Figures 2B–D**). Reliable performance of the trained algorithms was tested and validated on separate sets of randomly selected images. Raw data was processed and prepared for subsequent statistical analysis using RStudio and R v4.3.2 (34) with the packages phenoptr (35) and phenoptrReports (36). Immune cells were quantified by calculating cell densities (cells/mm^2^) and resultant proportions. For the DC subtypes as well as CD3^+^ and CD8^+^ T cells, the cell density was used for analysis, while functional CD8^+^ T cell phenotypes and T_H_ subtypes were analyzed as percentage of all CD8^+^ T cells and TF^+^ CD3^+^ T cells, respectively. Multi-channel TIFFs of representative images were processed by applying arithmetic point operations using the ImageJ software (37).

### 2.5 Statistical analysis

Immune cell infiltrates were assessed for the spatial compartments tumor stroma (TS), intraepithelial PanCK^+^ tumor (IET) and WTA, which combines both, TS and IET. To assess significant differences between immune cell frequencies and proportions of the TS and IET compartments, the Mann-Whitney-U test was used. For all analyses performed, p-values < 0.05 were considered statistically significant. Furthermore, immune cell infiltrates were compared between patient subgroups stratified by clinicopathological characteristics (tumor grading, p16 status, hypoxia) for the whole HNSCC cohort, as well as for HPV-, and hypoxia-associated subcohorts (clinical characteristics of subcohorts in **Supplementary Tables 3, 4**). Statistical differences between subgroups were assessed by performing the Mann-Whitney-U test. Resulting p-values were visualized with a heatmap using a color code from faint (non-significant) to intensely colored (significant, p-value < 0.05), with red representing a higher infiltration in tissues classified as grading 3 (G3), p16+, or hypoxia^high^ and blue encoding the opposite. As the heatmap gives no information about the data distribution, significant correlations were visualized as scatter dot plots (median with 95% confidence interval [CI]) separately. Potential clinical impact of HNSCC-infiltrating DCs, CD8^+^ T cells, and T_H_ subsets on OS, locoregional control (LRC), and control of locoregional recurrence and distant metastasis (LDMC) was explored for each of the three spatial compartments using Kaplan-Meier analysis with Log-rank test. Clinical endpoints OS, LRC, and LDMC were defined as the time interval between the first day of radiotherapy and the date of the event or last follow up (10, 30). The median density of each cell phenotype was used as cutoff between high (≥ median) and low infiltration (< median) subgroups. Log-rank p-values of Kaplan-Meier analysis were visualized using a heatmap that depicts the significant p-values (p < 0.05) in intense colors, where red represents that high infiltration improves the considered endpoint and blue encodes the opposite. For significant associations, Kaplan-Meier graphs are shown in addition to the heatmaps. Furthermore, immune cell populations and clinical parameters (tumor localization, p16 status, T stage) being significant in univariate setting were analyzed by multivariate Cox regression. All statistical analyses and visualization of the heatmaps were performed with Python 3.7.10 (Wilmington, Delaware, USA) using the lifelines and matplotlib packages. For visualization of scatter dot plots and Kaplan-Meier graphs, GraphPad Prism 9.4.1 (Boston, Massachusetts, USA) was used. Forest plots were created using the package forplo (38) with RStudio and R v4.3.2 (34).

## 3 Results

### 3.1 pDCs, T_regs_, PD-1^+^CD8^+^, and LAG-3^+^CD8^+^ T cells dominate immune landscape of HNSCC

**Figure 1:**
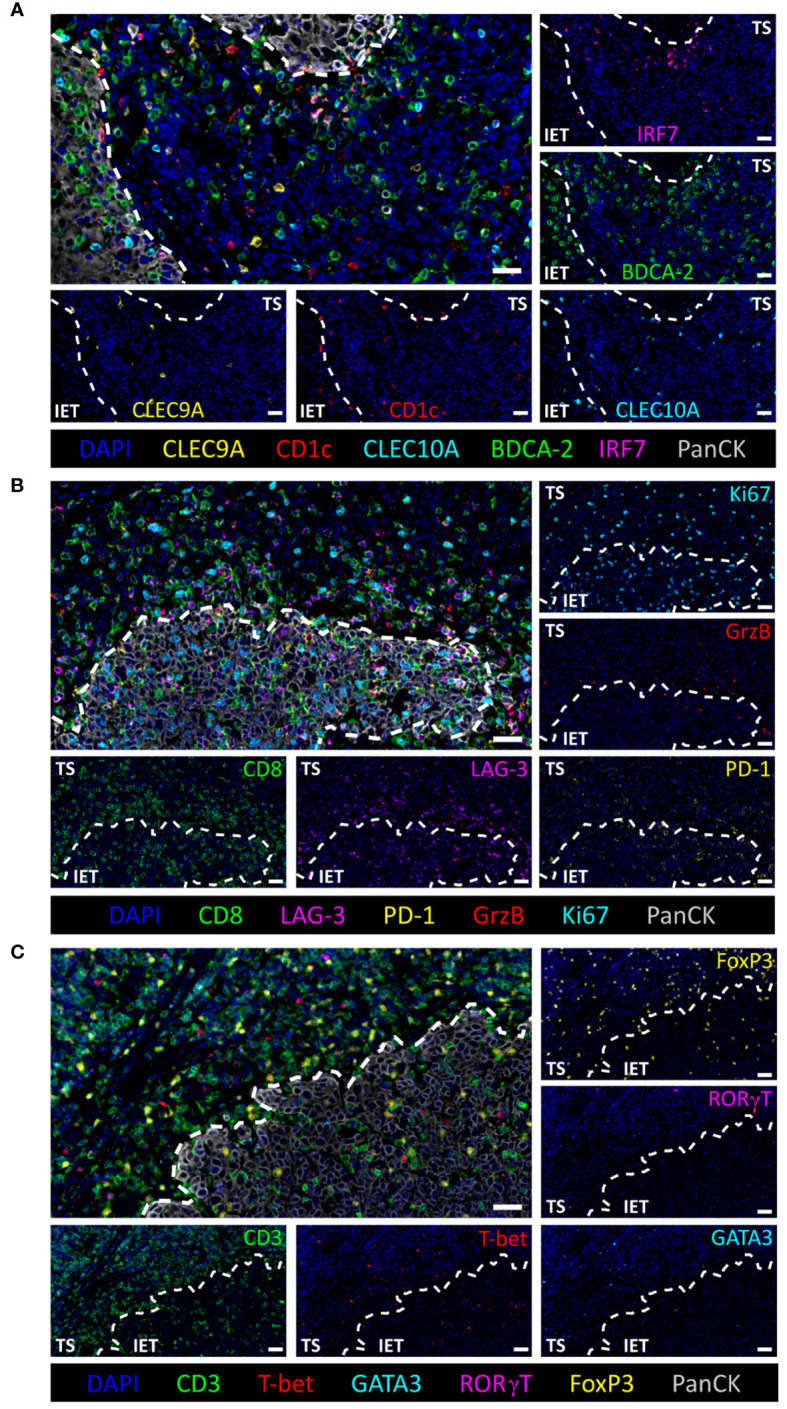
Multiparameter immunohistochemical stainings of tumor infiltrating immune cell populations. Representative images of **(A)** DCs (pDC: BDCA-2^+^, active pDC: IRF7^+^BDCA-2^+^, cDC1: CLEC9A^+^, cDC2: CLEC10A^+^CD1c^+^), **(B)** CD8^+^ T cell subtypes (inhibitory markers: LAG-3 and PD-1, cytotoxic marker: GrzB, proliferation marker: Ki67), and **(C)** T-helper cell subtypes (Treg: FoxP3^+^CD3^+^, T_H_1: T-bet^+^CD3^+^, T_H_2: GATA3^+^CD3^+^, T_H_17: RORγT^+^CD3^+^). Intraepithelial tumor cells and nuclei are visualized by PanCK and DAPI staining, respectively. White dashed line indicates separation of tumor stroma (TS) and intraepithelial tumor (IET) compartments. Scale bars indicate 30 µm.

For the phenotypic and spatial characterization of HNSCC-infiltrating immune cell populations, mIHC was applied to a cohort of 56 primary HNSCC patients that have undergone resection and adjuvant RTx. FFPE tissue slices were stained with three antibody panels to detect different DC subsets (cDC1, cDC2, pDCs), functional CD8^+^ T cell phenotypes (activated, proliferative, exhausted), and T_H_ subtypes based on the TFs T-bet (T_H_1 cells), GATA3 (T_H_2 cells), RORγT (T_H_17 cells), and FoxP3 (T_regs_). Representative images of these antibody panels are depicted in **Figures 1A–C**.

**Figure 2:**
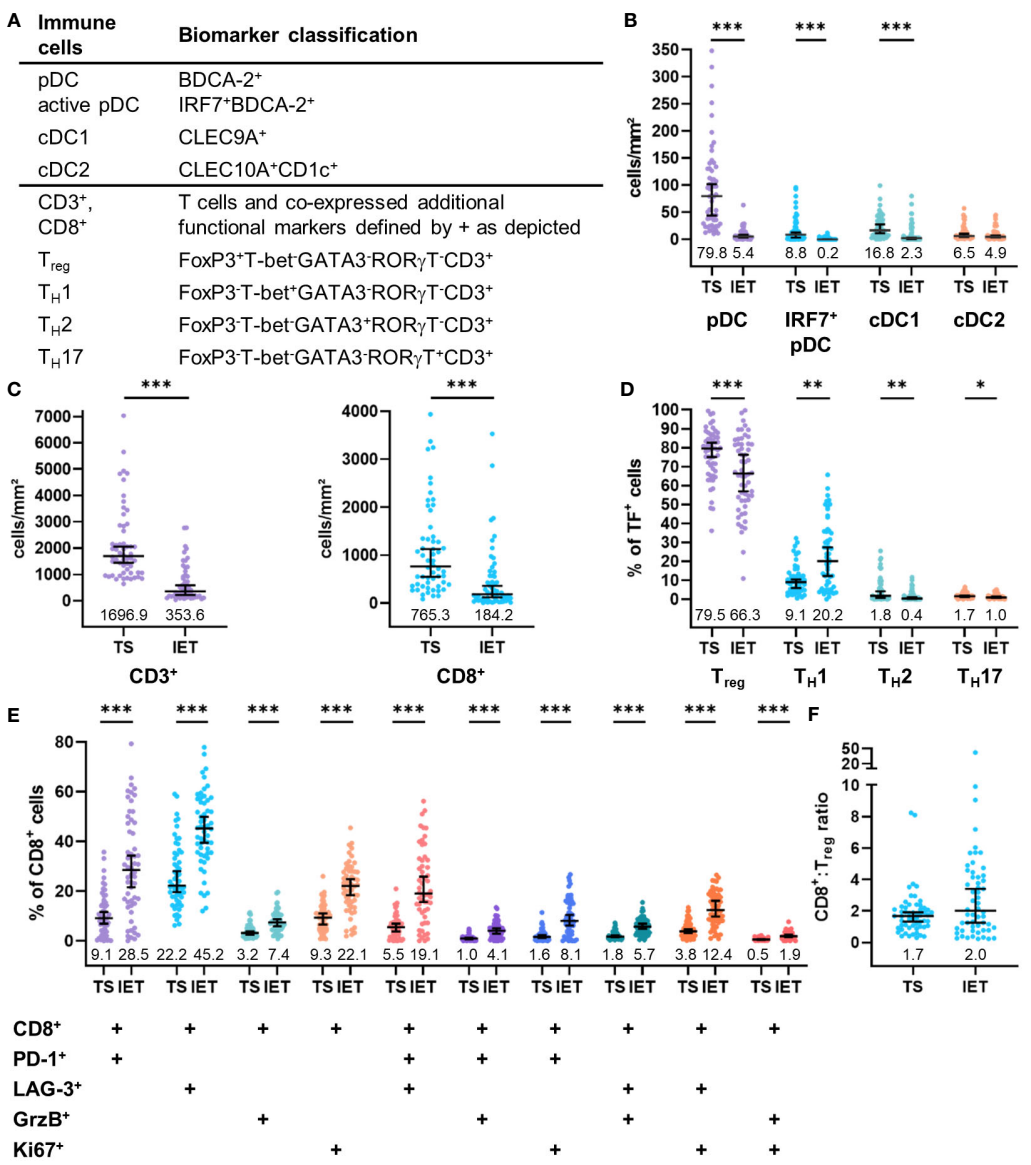
Quantified immune cell infiltration of tumor stroma (TS) and intraepithelial tumor (IET) compartments of the whole HNSCC cohort. **(A)** Biomarker classification used to identify several immune cells and subpopulations according to additional functional markers. Frequency of **(B)** DCs and **(C)** CD3^+^ (left) and CD8^+^ T cells (right). **(D)** Proportion of T_H_ subpopulations on total T cells expressing any transcription factor (TF). **(E)** Proportion of CD8 subpopulations expression further functional markers on total CD8^+^ T cells. Median with 95% confidence interval (CI), median values shown below dots, Mann-Whitney test, ***p < 0.001, **p < 0.01, *p < 0.05.

As the immunohistochemical staining allows to detect immune cells in their physical position within the original specimen, we compared the frequency of all infiltrated immune cell populations that are retrievable from the stained panels (**Figure 2A**) between the TS and the IET of all 56 HNSCC patients. The TS is defined as PanCK^-^ tissue regions in between the PanCK^+^ tumor bed termed as IET (**Figure 1**). Immune cell frequencies in the WTA (TS and IET combined) are shown in **Supplementary Figure 3**. In the DC population, pDCs represent the prevailing DC subtype in the WTA and the TS with a cell number of 39.1 cells/mm^2^ and 79.8 cells/mm^2^, respectively (**Figure 2B**; **Supplementary Figure 3**). Hence, pDCs were at least 4- or 6-times more abundant than cDC1 or cDC2. Comparing the densities of total pDCs, activated pDCs (nuclear IRF7^+^), and cDC1 between TS and IET, a significantly higher infiltration of the TS was observed for all three phenotypes, while cDC2 showed no difference between both tissue regions (**Figure 2B**). Furthermore, both CD3^+^ and CD8^+^ T cells were found in the TS (1696.9 CD3^+^ cells/mm^2^ and 765.3 CD8^+^ cells/mm^2^) in significantly higher numbers compared to the IET (353.6 CD3^+^ cells/mm^2^ and 184.2 CD8^+^ cells/mm^2^, **Figure 2C**). Regarding the T_H_ cell composition, T_regs_ accounted for the largest proportion of total TF^+^ CD3^+^ T cells in both tumor regions with 79.5% in the TS and 66.3% in the IET. Comparing the spatial distribution of T_H_ cells, a significantly higher proportion of T_H_1 cells was observed in the IET, while T_regs_, T_H_2 cells, and T_H_17 cells infiltrated the TS in a higher density (**Figure 2D**). Regarding functional CD8^+^ phenotypes, LAG-3^+^CD8^+^ T cells represented the highest proportion in both tumor regions with 22.2% in the TS and 45.2% in the IET (**Figure 2E**), followed by PD-1^+^CD8^+^, proliferative Ki67^+^CD8^+^, and potentially impaired/exhausted (PD-1^+^LAG-3^+^CD8^+^) T cells. We further examined the ratio of CD8^+^ T cells to T_regs_ with values > 1 indicating a higher CD8^+^ T cell infiltration. This ratio gives a simplified insight whether potential cytotoxic or immunosuppressive T cells prevail in the TME. However, we observed no significant difference between TS and IET while the CD8:T_reg_ ratio tended to be higher in the IET (**Figure 2F**). Taking together, the immunological TME of HNSCC is characterized by high frequencies of pDCs, T_regs_, and CD8^+^ T cells in the stromal compartment, whereby CD8^+^ T cells exhibited an exhausted phenotype (PD-1^+^ and/or LAG-3^+^) predominantly in the IET.

### 3.2 p16+ HNSCC exhibit high immune infiltration while hypoxic conditions reveal reduced immune cell infiltration

**Figure 3:**
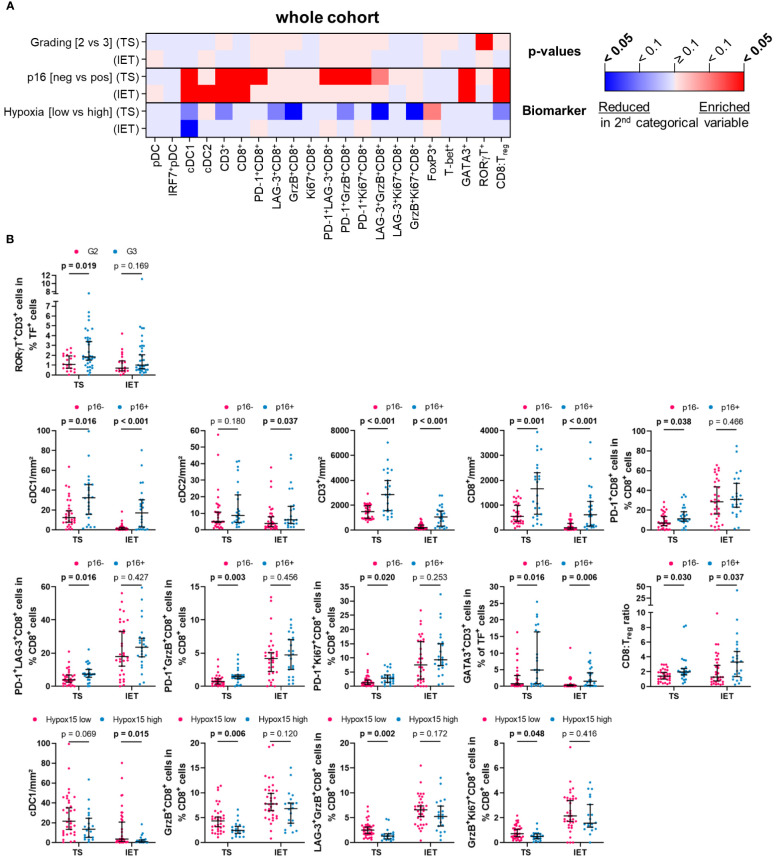
Quantified immune cell infiltration of tumor stroma (TS) and intraepithelial tumor (IET) compartments with respect to certain clinical parameters of the whole HNSCC cohort. **(A)** Calculated p-values (Mann-Whitney test) are depicted in the heatmap (red indicates higher immune cell frequencies in G3/p16+/Hypoxia high subcohorts; blue indicates higher immune cell frequencies in G2/p16-/Hypoxia low subcohorts; color intensity indicates significance level) and **(B)** in case of a significant difference in at least one compartment, TS and IET data were shown in separate dot plots (median with 95% confidence interval [CI], significant p-values [p < 0.05] printed in bold; TF, transcription factor).

**Figure 4:**
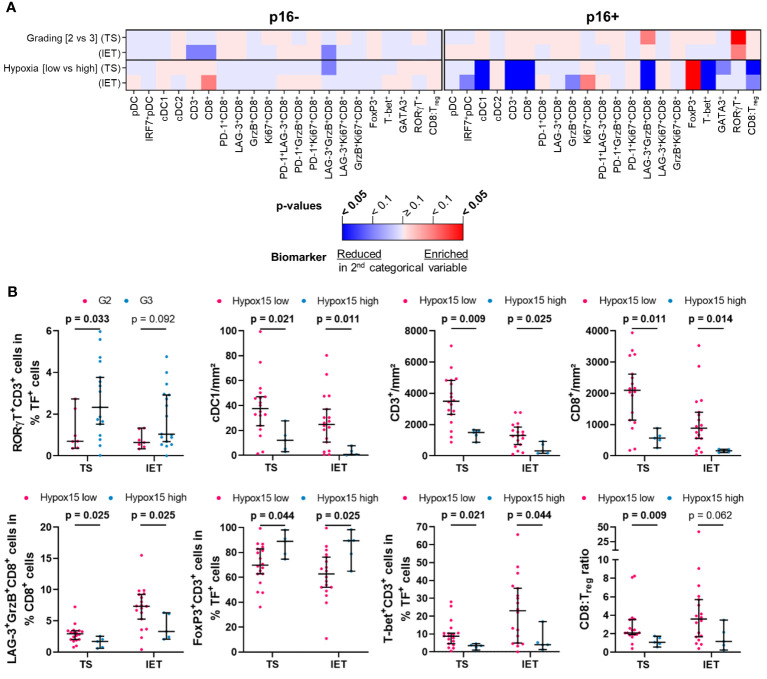
Quantified immune cell infiltration of tumor stroma (TS) and intraepithelial tumor (IET) compartments with respect to certain clinical parameters of p16+ and p16- subcohorts. **(A)** Calculated p-values (Mann-Whitney test) are depicted in the heatmap (red indicates higher immune cell frequencies in G3/Hypoxia high subcohorts; blue indicates higher immune cell frequencies in G2/Hypoxia low subcohorts; color intensity indicates significance level) and **(B)** in case of a significant difference in at least one compartment, TS and IET data were shown in separate dot plots (median with 95% confidence interval [CI], significant p-values [p < 0.05] printed in bold; TF, transcription factor).

**Figure 5:**
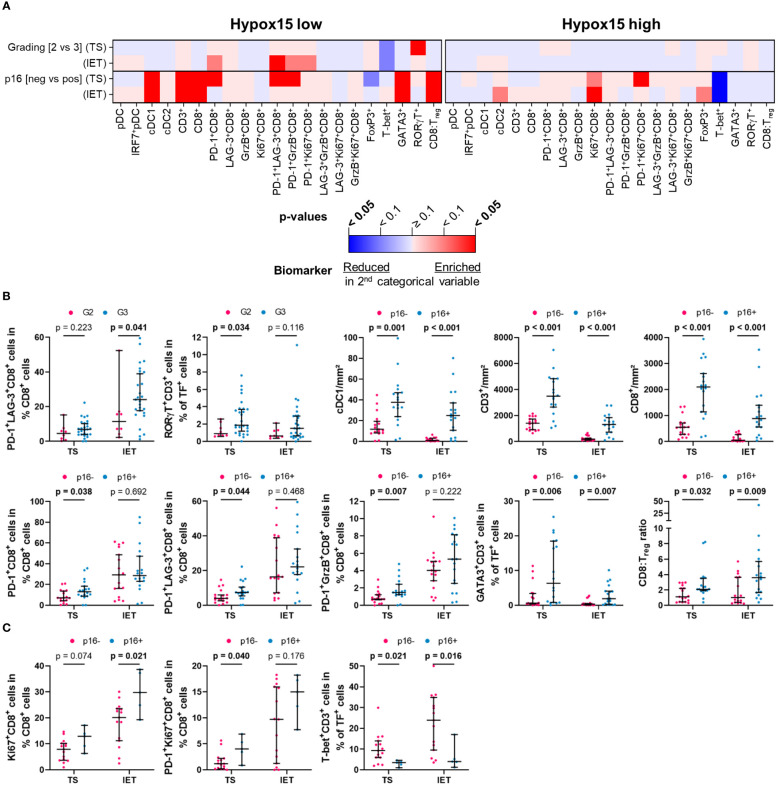
Quantified immune cell infiltration of tumor stroma (TS) and intraepithelial tumor (IET) compartments with respect to certain clinical parameters of Hypox15 high and Hypox15 low subcohorts. **(A)** Calculated p-values (Mann-Whitney test) are depicted in the heatmap (red indicates higher immune cell frequencies in G3/p16+ subcohorts; blue indicates higher immune cell frequencies in G2/p16- subcohorts; color intensity indicates significance level) and in case of a significant difference in at least one compartment, TS and IET data were shown in separate dot plots (median with 95% confidence interval [CI], significant p-values [p < 0.05] printed in bold) for **(B)** Hypox15 low and **(C)** Hypox15 high subcohorts (TF, transcription factor).

To assess a potential link between immune infiltrates and clinical parameters, we analyzed the frequency of HNSCC-infiltrating DCs and T cells with regard to tumor grading (G2 vs. G3), p16 positivity (p16 negative vs. p16 positive), and hypoxia (low vs. high expression of Hypox15 gene signature). We examined the whole cohort of 56 HNSCC patients (**Figure 3**) and additionally compared the p16-/p16+ subcohorts (**Figure 4**) and the Hypox15^low^/Hypox15^high^ subcohorts (**Figure 5**).

In the whole cohort, only T_H_17 cells (RORγT^+^CD3^+^) in the TS correlated positively with a higher tumor grading, which was not observed in the IET (**Figures 3A, B**). Regarding the biomarker analysis, p16 positivity was largely associated with a higher immune infiltration. In both locations, TS and IET, the density of cDC1, CD3^+^, and CD8^+^ T cells as well as the proportion of T_H_2 cells (GATA3^+^CD3^+^) and the CD8:T_reg_ ratio was significantly increased in p16+ HNSCC. In addition, several functional CD8^+^ T cell subpopulations showed a significantly higher proportion in the TS of p16+ tumors (PD-1^+^CD8^+^, PD-1^+^LAG-3^+^CD8^+^, PD-1^+^GrzB^+^CD8^+^, PD-1^+^Ki67^+^CD8^+^), while cDC2 were positively correlated to p16 overexpression only in the IET (**Figures 3A, B**). A high expression of hypoxia-related genes was associated with a significantly decreased cDC1 infiltration in the IET and a significantly smaller proportion of functional CD8^+^ subtypes (GrzB^+^CD8^+^, LAG-3^+^GrzB^+^ CD8^+^, GrzB^+^Ki67^+^CD8^+^) in the TS (**Figures 3A, B**).

Analyzing the p16+ and the p16- subcohorts, we found that the clinicopathological parameters tumor grading or hypoxia status seem to have no significant effect on the frequencies of DCs and T cells in the TS and IET compartments of p16- tumors (**Figure 4A**). In contrast, in the WTA of the p16- subcohort, activated GrzB^+^CD8^+^ and proliferating Ki67^+^CD8^+^ T cells with or without the expression of LAG-3 showed a significantly lower proportion in Hypox15^high^ tumors (**Supplementary Figure 5**). In the p16+ subcohort, a significantly higher proportion of T_H_17 cells (RORγT^+^CD3^+^) was observed in the TS of G3 compared to G2 HNSCC (**Figures 4A, B**). Additionally, a high hypoxia-related gene expression was associated with less infiltrates of cDC1, CD3^+^, and CD8^+^ T cells as well as a decreased proportion of LAG-3^+^GrzB^+^CD8^+^ T cells in both tumor areas (**Figures 4A, B**). Interestingly, under hypoxic conditions, p16+ tumors exhibited a significantly decreased proportion of T_H_1 cells (T-bet^+^CD3^+^) combined with increased numbers of immunosuppressive T_regs_ (FoxP3^+^CD3^+^). In line with this observation, the CD8:T_reg_ ratio was lower in Hypox15^high^ tumors (**Figures 4A, B**).

Comparing the hypoxia-stratified subcohorts, clinicopathological parameters (tumor grading and p16 status) had a greater influence on the immune cell infiltrate in tumors with a low compared to tumors with a high Hypox15 gene cluster expression (**Figure 5A**). While tumor grading had no significant effect in the Hypox15^high^ subcohort, G3 HNSCC were significantly associated with increased proportions of PD-1^+^LAG-3^+^CD8^+^ T cells in the IET and T_H_17 cells (RORγT^+^CD3^+^) in the TS of the Hypox15^low^ cohort (**Figures 5A, B**). Furthermore, Hypox15^low^ p16+ tumors exhibited an increased infiltration of cDC1, CD3^+^, and CD8^+^ T cells and had significantly higher proportions of PD-1^+^CD8^+^, PD-1^+^LAG-3^+^CD8^+^, and PD-1^+^GrzB^+^CD8^+^ T cells in the TS. Additionally, these tumors were characterized by high proportions of T_H_2 cells (GATA3^+^CD3^+^) and a high CD8:T_reg_ ratio (**Figures 5A, B**). In contrast, Hypox15^high^ p16+ tumors exhibited a significantly decreased amount of T_H_1 cells (T-bet^+^CD3^+^), while few functional CD8^+^ phenotypes showed significantly higher values in these tumors (Ki67^+^CD8^+^ T cells in IET, PD-1^+^Ki67^+^CD8^+^ T cells in TS, **Figures 5A, C**). In general, p16 positivity was associated with increased frequencies of several DC and T cell subpopulations, whereas hypoxia correlated with opposite effects. These infiltration patterns were not only recognizable in the whole cohort but also in p16+ and Hypox15^low^ subcohorts.

### 3.3 Prognostic association of immune cells is dependent on biomarker subgrouping

**Figure 6:**
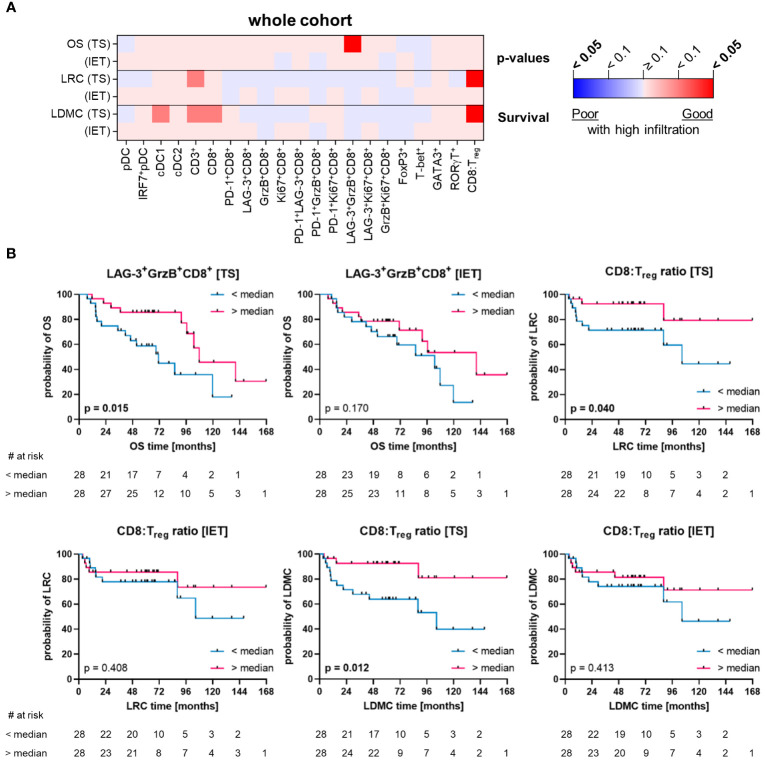
Quantified immune cell infiltration of tumor stroma (TS) and intraepithelial tumor (IET) compartments with respect to survival parameters of the whole HNSCC cohort. **(A)** Calculated p-values (Log-rank test) are depicted in the heatmap (red indicates a positive association of survival probabilities with tumor infiltration; blue indicates a negative association of survival probabilities with tumor infiltration; color intensity indicates significance level) and **(B)** in case of a significant difference in at least one compartment, TS and IET data were shown in separate Kaplan-Meier curves (significant p-values [p < 0.05] printed in bold).

**Figure 7:**
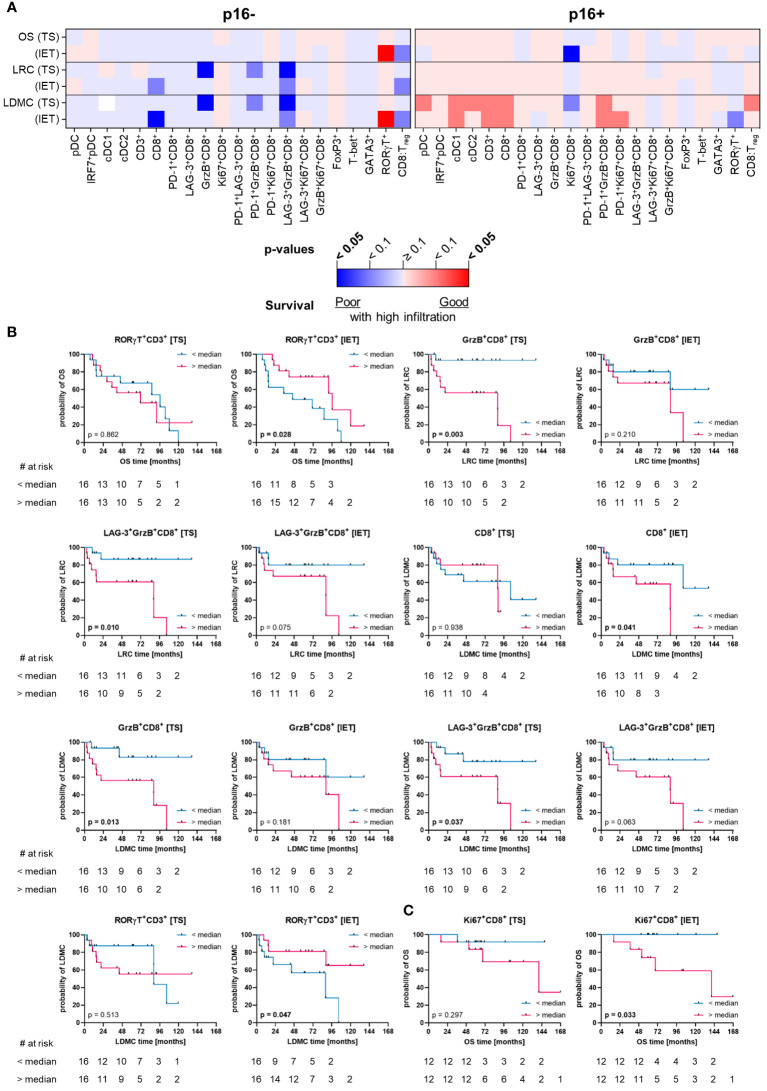
Quantified immune cell infiltration of tumor stroma (TS) and intraepithelial tumor (IET) compartments with respect to survival parameters of p16+ and p16- subcohorts. **(A)** Calculated p-values (Log-rank test) are depicted in the heatmap (red indicates a positive association of survival probabilities with tumor infiltration; blue indicates a negative association of survival probabilities with tumor infiltration; color intensity indicates significance level) and in case of a significant difference in at least one compartment, TS and IET data were shown in separate Kaplan-Meier curves for **(B)** p16- and **(C)** p16+ subcohorts (significant p-values [p < 0.05] printed in bold).

**Figure 8:**
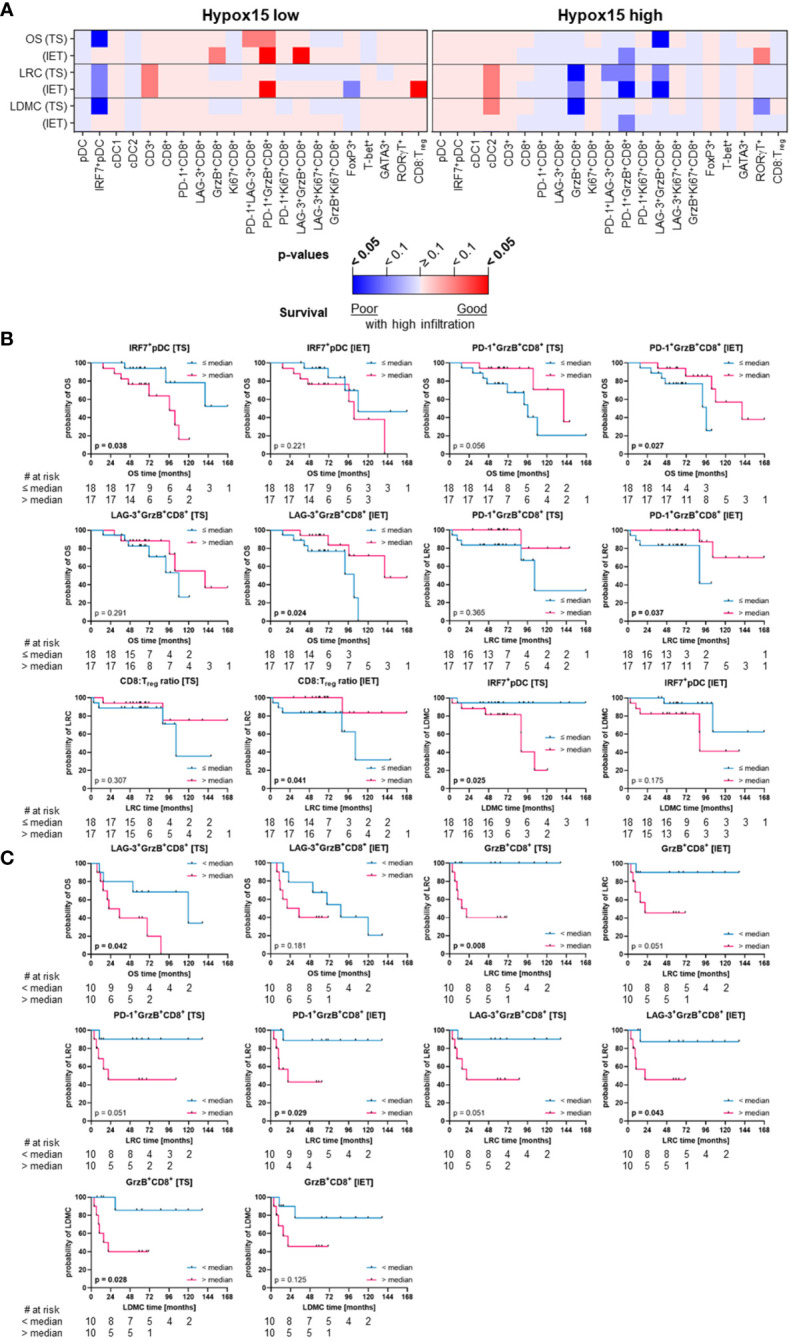
Quantified immune cell infiltration of tumor stroma (TS) and intraepithelial tumor (IET) compartments with respect to survival parameters of Hypox15 high and Hypox15 low subcohorts. **(A)** Calculated p-values (Log-rank test) are depicted in the heatmap (red indicates a positive association of survival probabilities with tumor infiltration; blue indicates a negative association of survival probabilities with tumor infiltration; color intensity indicates significance level) and in case of a significant difference in at least one compartment, TS and IET data were shown in Kaplan-Meier curves for **(B)** Hypox15 low and **(C)** Hypox15 high subcohorts (significant p-values [p < 0.05] printed in bold).

To specifically determine the influence of HNSCC-infiltrating DC and T cell subpopulations on clinical outcome, we evaluated different clinical parameters (OS, LRC, LDMC) in the whole cohort of 56 HNSCC patients (**Figure 6**) as well as in subcohorts stratified by p16 overexpression and hypoxia-related gene expression (**Figures 7**, **8**).

In the whole cohort, a significant positive correlation with OS was observed only for LAG-3^+^GrzB^+^CD8^+^ T cells (**Figures 6A, B**). In addition, a high CD8:T_reg_ ratio was associated with a decreased risk of local recurrence and distant metastases (LRC and LDMC) of HNSCC (**Figures 6A, B**). Of note, these prognostic correlations were only observed in the TS, while immune cells in the IET had no prognostic value in the whole cohort (**Figure 6A**). In contrast, when considering TS and IET compartments together (WTA), high infiltration of T cells (CD3^+^ and CD8^+^) as well as cDC1 were significantly associated with a longer recurrence-free period (LRC and LDMC, **Supplementary Figure 4**). Moreover, high numbers of PD-1^+^LAG-3^+^CD8^+^ T cells correlated significantly with a longer OS (**Supplementary Figure 4**).

p16+ HNSCC and tumors located in the oropharynx are generally associated with better treatment response (8, 9). The comparison of p16- and p16+ subcohorts revealed that the analyzed immune cells in TS or IET compartments of the p16+ cohort had less significant prognostic associations than the p16- cohort (**Figure 7A**). In the p16- cohort, HNSCC with high CD8^+^ T cell infiltrates in the IET displayed significantly lower LDMC, which was observed as a trend for LRC (**Figures 7A, B**). Furthermore, p16- HNSCC with a high stromal proportion of activated GrzB^+^CD8^+^ T cells and LAG-3^+^GrzB^+^CD8^+^ T cells showed a significant negative correlation with disease progression (LRC and LDMC) and a higher frequency of T_H_17 cells (RORγT^+^CD3^+^) was associated with longer OS and LDMC in these tumors (**Figures 7A, B**). In addition, T_regs_ also showed a positive correlation with longer OS in the WTA (**Supplementary Figure 5**). In contrast, in p16+ HNSCC only increased proportions of proliferating Ki67^+^CD8^+^ T cells in the IET were significantly associated with shorter OS (**Figures 7A, C**).

Regarding the hypoxia-subcohorts, a high frequency of activated pDCs (IRF7^+^ pDCs) in the TS was significantly associated with a shorter OS and LDMC in the Hypox15^low^ subcohort (**Figures 8A, B**). Additionally, Hypox15^low^ tumors with high proportions of PD-1^+^GrzB^+^CD8^+^ T cells and LAG-3^+^GrzB^+^ T cells in the IET showed positive outcomes regarding OS/LRC and OS, respectively (**Figures 8A, B**). Moreover, a high CD8:T_reg_ ratio in the IET compartment of these tumors was indicative for a significantly higher LRC (**Figures 8A, B**), which was also observed when analyzing the WTA (**Supplementary Figure 6**). Likewise, the negative association between activated pDCs (IRF7^+^ pDCs) and LDMC persist for the WTA, while also high pDC numbers were significantly associated with a shorter OS (**Supplementary Figure 6**). In contrast, high cDC1, CD3^+^, and CD8^+^ T cell infiltration were prognostic for a significantly longer recurrence-free period in the WTA of Hypox15^low^ tumors (LRC and LDMC, **Supplementary Figure 6**). In Hypox15^high^ HNSCC, several CD8^+^ T cell phenotypes were significantly associated with worse prognosis. A high proportion of activated GrzB^+^CD8^+^ T cells in the TS was associated with a higher risk of recurrence (LRC and LDMC, **Figures 8A, C**). For LRC, this was also observed for PD-1^+^GrzB^+^ T cells and LAG-3^+^GrzB^+^CD8^+^ T cells in the IET. Only LAG-3^+^GrzB^+^CD8^+^ T cells in the TS were additionally predictive for shorter OS in Hypox15^high^ cohort (**Figures 8A, C**). In addition to T cells, high frequencies of cDC2 were predictive of a high LDMC when analyzing the whole tumor of Hypox15^high^ HNSCC (**Supplementary Figure 6**).

**Figure 9:**
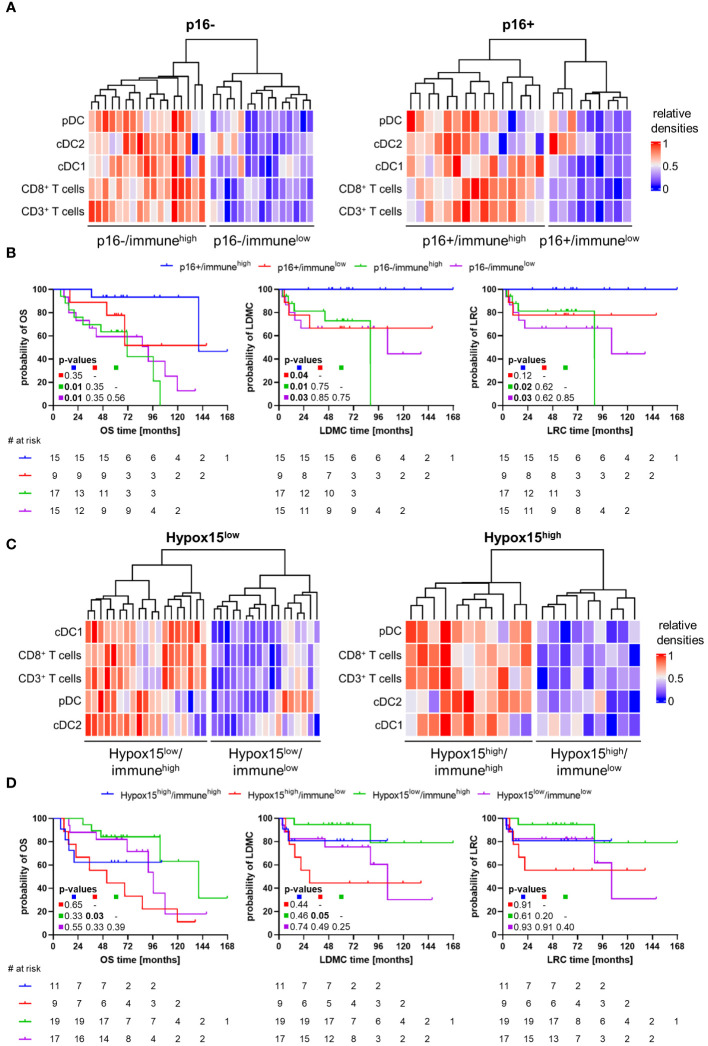
Combined p16/immune- and Hypox15/immune-phenotypes for HNSCC patient stratification in terms of clinical outcome. **(A)** Heatmaps of p16- (left) and p16+ (right) subcohorts clustering in two groups each according to low (blue) or high (red) immune cell infiltration by pDCs, cDC1, cDC2, CD3^+^, and CD8^+^ T cells. **(B)** Kaplan-Meier curves depict clinical outcome (OS, LRC, LDMC) for HNSCC patients with p16+/immune^high^, p16+/immune^low^, p16-/immune^high^, or p16-/immune^low^ phenotypes. **(C)** Heatmaps of Hypox15^low^ (left) and Hypox15^high^ (right) subcohorts clustering in two groups each according to low (blue) or high (red) immune cell infiltration by pDCs, cDC1, cDC2, CD3^+^, and CD8^+^ T cells. **(D)** Kaplan-Meier curves depict clinical outcome (OS, LRC, LDMC) for HNSCC patients with Hypox15^high^/immune^high^, Hypox15^high^/immune^low^, Hypox15^low^/immune^high^, or Hypox15^low^/immune^low^ phenotypes. p-values calculated by Log-rank test and adjusted using Benjamini-Hochberg method, significant p-values shown in bold print.

After analyzing whether p16 or hypoxia status of HNSCC patients has an influence on the infiltration of individual immune cells in several tumor compartments and whether this has an impact on the clinical outcome, the influence of several DC subsets in combination with T cells on patient survival was examined. This allows a more detailed patient classification and provides possible starting points for further research aiming at novel therapeutic approaches. Therefore, we clustered the subcohorts stratified by p16 positivity and hypoxia-related gene expression separately according to high or low immune cell infiltrate generating four different clusters for each subcohort (**Figures 9A, C**). In order to encompass several immune cell types, the clustering was based on densities of pDCs, cDC1, cDC2, CD3^+^, and CD8^+^ T cells in WTA (TS and IET together). Patients with p16+ HNSCC having significantly improved clinical outcome compared to patients with p16- tumors (**Supplementary Figure 7**), showed these favorable clinical results especially in combination with high immune cell infiltrate (**Figure 9B**). In comparison to both p16- clusters, significant differences were visible for p16+/immune^high^, but not for p16+/immune^low^. In terms of combined hypoxia/immune phenotypes, it became obvious that particularly patients with Hypox15^low^ tumors and a high immune cell infiltrate depict the best survival and, in turn, patients with Hypox15^high^/immune^low^-classified tumors showed the worst outcomes (**Figure 9D**). Although patients with Hypox15^low^ tumors have a significantly prolonged OS compared to patients with Hypox15^high^ tumors (**Supplementary Figure 7**), the additional inclusion of immune cell infiltrate for further subclassification shows a trend towards improved clinical outcome by high immune cell infiltrate.

### 3.4 cDC1 and CD8^+^ T cells independently predict outcome of HNSCC patients

**Figure 10:**
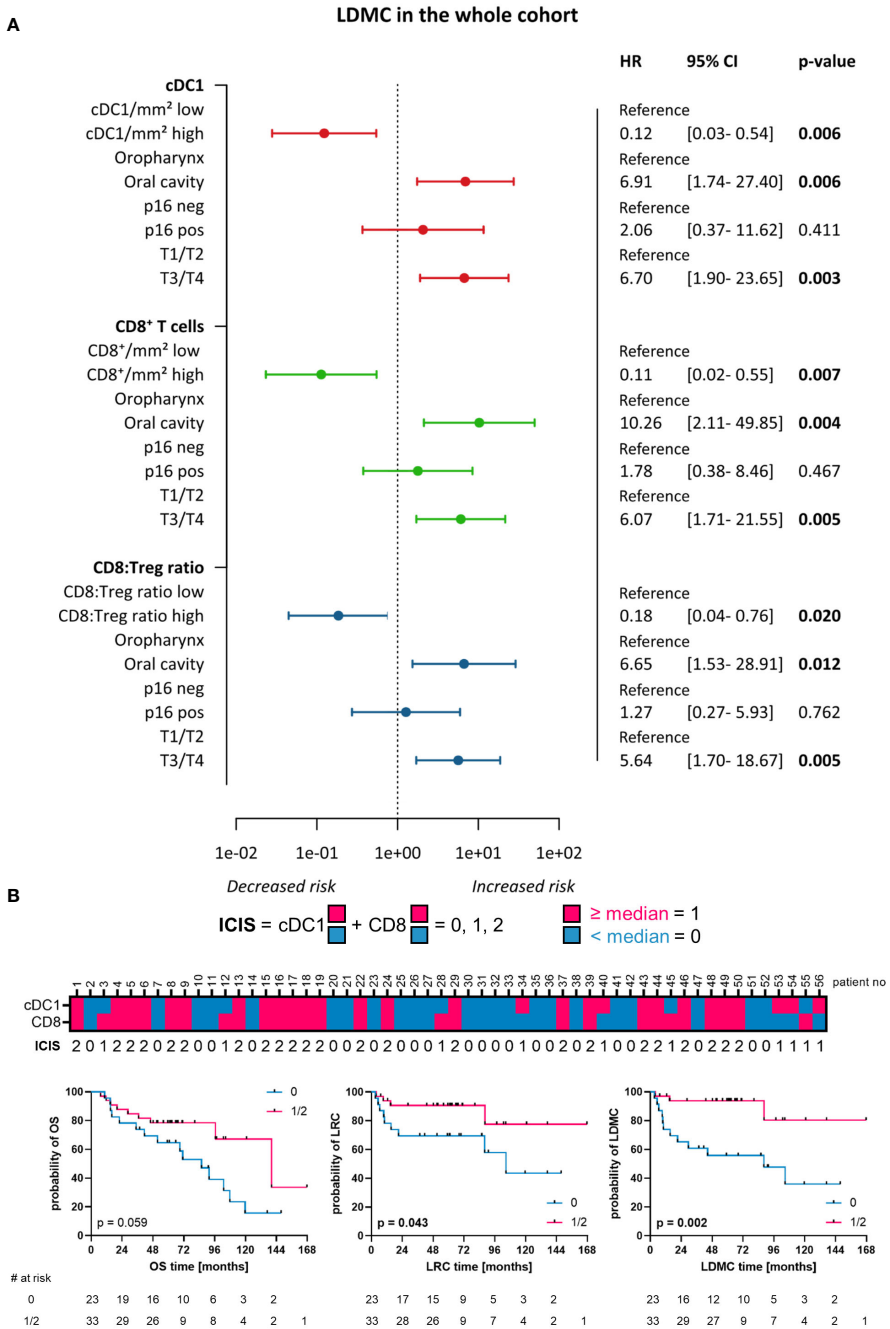
Multivariate Cox Regression and immune cell infiltration score (ICIS). **(A)** Forest plots visualizing Cox regression for cDC1, CD8^+^ T-cells, and CD8:Treg ratio with respect to LDMC and clinicopathological characteristics of the whole HNSCC cohort (localization, p16 positivity, T stage). Hazard ratio (HR) with 95% CI (confidence Interval). Significant p-values (p < 0.05) printed in bold. **(B)** ICIS combining high (≥ median) or low (< median) infiltration of cDC1 and CD8^+^ immune cells. Heatmap shows ICIS for each patient included within the study and Kaplan-Meier curves depict clinical outcome (OS, LRC, LDMC) for patients with ICIS = 0 vs. ICIS = 1 or ICIS = 2 (p-values calculated by Log-rank test, significant p-values [p < 0.05] printed in bold).

Additionally, DC and T cell subpopulations were tested in a multivariate setting to obtain immune markers that can serve as independent prognostic factors for HNSCC. Therefore, Cox regression was used to evaluate the prognosis of infiltrating immune cells in relation to different clinical parameters (**Figure 10A**; **Supplementary Figures 8, 9**; **Supplementary Table 5**). The clinical parameters and immune cell subtypes used for multivariate analysis were chosen based on a significant prognosis in the univariate setting. As the establishment of new prognostic biomarkers should be easily transferable into clinical procedures, we performed the multivariate analysis for the WTA as a differentiation into TS and IET may not be feasible in routine diagnostics. We observed that a high density of cDC1 is a prognostic factor for higher LRC and LDMC in the whole cohort, independent of tumor location (oropharynx vs. oral cavity), p16 positivity, and tumor stage (T1/T2 vs. T3/T4 according to TNM staging, **Figure 10A**; **Supplementary Figure 8**). Analyzing the T cell subpopulations, a high density of CD8^+^ T cells was independently associated with a longer recurrence-free period (LRC and LDMC) in the whole HNSCC cohort (**Figure 10A**; **Supplementary Figure 8**). Furthermore, the proportion of activated GrzB^+^CD8^+^ T cells was significantly associated with a higher risk of recurrence (LRC and LDMC) in patients with p16- HNSCC, independent of tumor staging (**Supplementary Figure 9**). In HNSCC with high hypoxia-related gene expression, a high proportion of PD-1^+^GrzB^+^CD8^+^ T cells correlated significantly with a shorter OS and LDMC, independent of tumor location and tumor staging (**Supplementary Figure 9**). In addition, the ratio of CD8^+^ T cells to T_regs_ seemed to has an independent prognostic value, as a higher CD8:T_reg_ ratio was significantly associated with a decreased risk of local and distant recurrence (LDMC) in the whole cohort and the Hypox15^low^ subcohort (**Figure 10A**; **Supplementary Figure 9**).

Since two immune cell types, in particular cDC1 and CD8^+^ T cells, stood out in their favorable prognosis for the whole HNSCC cohort, we developed an immune cell infiltration score (ICIS) based on the frequency of both cell types in the WTA (**Figure 10B**). To generate the ICIS, a binary code was applied to each cell type that was based on the median cell density (density ≥ median = 1, density < median = 0). By summarizing the binary code for both cell types, a classification system was obtained that consisted of three stages: 0 – cell density of both markers is below median, 1 – cell density of only one marker is above median, 2 – both cell densities are above median. When stratifying the patient cohort according to the ICIS (0 vs. 1/2) we observed that an ICIS of 1 to 2, reflecting a high infiltration of cDC1 and/or CD8^+^ T cells, was significantly associated with a higher LRC and highly significant in predicting a high LDMC (**Figure 10B**). Furthermore, a higher ICIS showed a strong trend for the prediction of prolonged OS (**Figure 10B**).

## 4 Discussion

The significant influence of the immune landscape on tumor development, progression, as well as on therapeutic outcome, has been described for many solid cancers, including HNSCC. By using conventional hematoxylin and eosin (H&E) whole slide stainings, Ribbat-Idle et al. showed that the immune infiltration pattern is an independent prognostic factor for OS of HNSCC patients, with “cold” tumors being significantly associated with worse clinical outcome in comparison to “hot” and “excluded” tumors (39). Whereas CD8^+^ T cell infiltration in general indicates a positive prognosis in HNSCC, T_H_ subpopulations, DC subtypes, and CD8^+^ T cells expressing further co-stimulatory/-inhibitory marker proteins lack prognostic evaluation, so far (40, 41).

In our study, we investigated the tumor infiltration pattern of several immune cell subpopulations in different tumor compartments. Regarding DC subtypes, predominant pDCs were significantly more abundant in TS than in IET tissue compartment, whereby only minor frequencies of mature and/or activated pDCs (IRF7^+^ pDCs) were identified. In general, HNSCC samples exhibited eightfold higher pDC infiltration in comparison to primary resected colon cancer and pancreatic ductal adenocarcinoma samples in our previous studies using similar BDCA-2 staining procedures (23, 42). Especially in HNSCC, the hypoxic TME seems to inhibit pDC maturation (43). Koucký et al. demonstrated a colocalization of functionally impaired pDCs with T_regs_ in TS and reported a selective pDC-fostered T_reg_ expansion (44). When looking at T cell subpopulations in our HNSCC cohort, T_regs_ represented the major T_H_ cell subset, with their predominant stromal distribution confirming current literature (45) and may contributing to the mentioned relation of pDCs and T_regs_. With respect to further T_H_ subsets, T_H_1 cells were more common than T_H_2 cells or T_H_17 cells and significantly more frequent in IET than in TS. Although the development of HNSCC is thought to be accompanied by a switch from T_H_1- to T_H_2-cytokines (46), a CD8^+^-rich TME seems to be linked to a T_H_1-dominated immune response (47). Using The Cancer Genome Atlas (TCGA) datasets of HNSCC patients, Lu et al. found an increased level of T_H_1 cells in a subcohort with a high abundance of γδT cells, being in turn associated with a longer OS (48). Due to the limited number of marker molecules that can be simultaneously analyzed in one staining panel using our mIHC method, we have chosen CD3 for T cell detection and various major TFs (T-bet, GATA3, RORγT, and FoxP3) for evaluating the proportion of the most common T_H_ subsets (T_H_1 cells, T_H_2 cells, T_H_17 cells, T_regs_). Therefore, rare CD8^+^ T cells (T_C_1, T_C_2, T_C_17) expressing one of these TFs may contribute to the proportion of positively stained TF-expressing CD3^+^ T cells in our study. In this context, T_C_1, T_C_2, and T_C_17 cells have been found in peripheral blood (49) or tumor draining lymph nodes (50) of HNSCC patients by flow cytometry. These studies discriminated the subpopulations based on the cytokine expression profiles without analyzing TF expression. However, CD8^+^ T_C_ subsets were less frequent than CD4^+^ T_H_ subsets. In addition to αβT cells, γδT cells expressing the mentioned TFs may also make up a proportion of our positively stained CD3^+^ T cells. IL17-producing γδT cells, termed γδT17, were found in several tumor entities, including HNSCC (51, 52). In further studies, we explored the frequency and phenotype of HNSCC-infiltrating T cells. In comparison to a previous study on soft tissue sarcomas, that utilized a similar T cell staining procedure, HNSCC samples featured an obviously higher T cell infiltration (32). The investigated CD8^+^ T cells showed significantly higher frequencies in TS. This finding is in line with the literature reporting a higher frequencies of stromal T lymphocytes compared to IET-lying ones (53). However, significantly greater proportions of CD8^+^ T cell subpopulations, expressing further co-stimulatory and/or co-inhibitory marker proteins, were present in the IET compartment. In particular, a high expression of the co-inhibitory receptors PD-1 and LAG-3 by CD8^+^ T cells, either alone or in combination, became obvious underlining the described loss of effector function and exhaustion state of tumor-infiltrated CD8^+^ T cells (5).

Following previous approaches, we incorporated several clinicopathological parameters in our analysis that appear important for the progression of HNSCC and treatment response, such as tumor grading, p16 status, and hypoxia, and assessed them in conjunction with immune cell infiltrate patterns. First, we explored the relationship between the immune landscape and tumor grading. While Boucek et al. described a positive association between tumor histological grading and elevated CD8^+^ T cell levels in the blood of HNSCC patients at diagnosis (54), we observed a positive correlation between tumor grading and exhausted CD8^+^ T cells (PD-1^+^LAG-3^+^CD8^+^) within the IET compartment of the Hypox15^low^ subcohort. This finding is in line with the literature describing a correlation of high LAG-3 expression and high pathological grade (55). Further, a higher grading (G3) was frequently accompanied by a higher proportion of stromal RORγT^+^ T_H_17 cells. A high infiltration of T_H_17 cells in HNSCC was confirmed by others, hypothesizing on a contribution to cancer progression (21).

Growing evidence has shown that two factors, namely tumor localization and HPV status, split HNSCC into greatly different subentities, characterized by distinct clinical and therapeutic outcomes (7). Recognition of HPV-related HNSCC as a distinct entity from the HPV-unrelated cases emerged from differences observed in their molecular profiles, tumor development process, clinical presentation, and outcome. This led to a novel staging system for the HPV+ oropharyngeal squamous cell carcinoma in the latest edition of the TNM classification of the Union for International Cancer Control (UICC) (56). The immune contexture, which differs among HNSCC localizations and between HPV+ and HPV- tumors, might represent a pivotal player in shaping these differences. Multiple groups have reported an enhanced infiltration of CD8^+^ T lymphocytes in p16+ HNSCC, that correlated positively with clinical outcome (14, 24, 47, 57, 58). In addition to high CD3^+^ and CD8^+^ T cell densities as well as CD8:T_reg_ ratio in HPV+ tumors shaping a less immunosuppressive TME as detected in multiple studies (41), we showed that both cDC subtypes (cDC1 and cDC2) had significantly higher frequencies in p16+ vs. p16- tumor samples, primarily in the IET compartment. Similarly, researchers reported a higher infiltration of HPV+ oropharyngeal tumors by a specific CD11c^+^ cDC population compared to the HPV- counterparts (59). Using the TCGA database, Gameiro et al. observed that activated DC transcript levels tend to be higher in HPV+ vs. HPV- tumors, but without reaching significance (60). However, also contradictory results are reported being not able to draw a link between DC frequencies and HPV status or even detecting lower DC amounts in HPV-related tumors (41, 59). Additionally, increased proportions of exhausted T cells (PD-1^+^CD8^+^, PD-1^+^LAG-3^+^CD8^+^), cytotoxic T cells (PD-1^+^GrzB^+^CD8^+^), proliferating T cells (PD-1^+^Ki67^+^CD8^+^), and GATA3^+^ T_H_2 cells in TS of patients with p16+ tumors were observed. Similarly to our quantified stainings, HPV-related HNSCC are ascertained to exhibit high expression levels of checkpoint receptors, including PD-1 and LAG-3, underlining a linkage of HPV infection and T cell exhaustion (53, 61, 62). Further, Kansy et al. reported significantly increased fractions of cytotoxic T cells (GrzB^+^CD8^+^) and proliferating T cells (Ki67^+^CD8^+^) in the TS of 27 HPV+ oropharyngeal tumor patients analyzed by multi-color immunofluorescence (63).

Hypoxia is a major factor that shapes the TME and also the response of HNSCC to radiotherapy by regulating i) the survival, proliferation, differentiation, and antitumor capacity of T cells; ii) the survival, migration, antigen presentation, and differentiation of DCs; or iii) the macrophage polarization and tumor development, among others (64, 65). In HNSCC, Kim et al. observed that the level of hypoxia varies across anatomical locations, with tumors in the larynx being less hypoxic than those in the oral cavity, and those in the oropharynx being the most hypoxic ones (66). In our HNSCC cohort, significant lower infiltration of cDC1 cells (IET) and cytotoxic GrzB^+^CD8^+^ T cells (TS) in tumors with hypoxia-associated gene signatures was measurable. According to the literature, hypoxia might diminish the infiltration and activity of DCs as well as T lymphocytes (67, 68) confirming our results. Especially in the p16+ subcohort, additional significant differences were noticeable, as these patients exhibited also a lower proportion of T-bet^+^ T_H_1 cells and a concomitantly higher infiltration of T_regs_ in Hypox15^high^ tumors being independent of the investigated tumor compartment. It is reported that immunosuppressive immune cells, like T_regs_, infiltrate hypoxic regions in HNSCC supporting our results (69).

Besides correlating immune cell infiltrate patterns with clinical parameters, the association of immune cell populations and patient survival was a major research objective of this study. By means of TCGA datasets, Mito et al. showed a lymphocyte gene expression signature that predicts prolonged OS, whereas a myeloid/DC signature indicated lower survival propabilites (5). This suggests that the myeloid compartment favors immune suppression and tumor growth in HNSCC, but its role has not been clearly dissected so far. In our cohort, patients with Hypox15^low^ HNSCC and a high stromal infiltration of IRF7^+^ pDCs displayed significantly shortened OS and LDMC. The correlation of pDC abundancy and poor prognosis is described for many cancer entities including HNSCC (41, 43). In other studies, pDC infiltration correlated with a high expression of FoxP3 and co-inhibitory markers (PD-1 and LAG-3) setting up an immunosuppressive TME and promoting tumor progression (70, 71). Additionally, positive correlations of especially cDC1 infiltration and, to a lesser extent, cDC2 frequency with favorable LDMC surfaced in our investigation. This fits and adds to the existing knowledge on cDC1s forming clusters with CD8^+^ T cells in the TS compartment of HNSCC patients and, thus, being capable to induce CD8^+^ T cell-mediated antitumoral responses, which in turn correlated with high LDMC in these patients (72). However, we report the positive role of cDC1s in HNSCC by detecting the subset-specific CLEC9A molecule at protein level. Our findings on cDC2s are in line with current literature describing an association of cytokine-producing CD163^+^ cDC2 cells or cDC2, expressing programmed cell death ligand-1 (PD-L1)^high^ and inducible T cell co-stimulator ligand (ICOSL)^low^, with favorable survival (73, 74).

Regarding an association of T cell subpopulations with clinical outcome, studies resulted in contradictory findings. On the one hand, CD4^+^ T cells were significantly associated with longer OS and disease-specific survival (75), but on the other hand most research did not observe significant correlations of CD4^+^ infiltration and patient prognosis (41, 61). In our p16- subcohort, the infiltration of RORγT^+^ T_H_17 cells and FoxP3^+^ T_regs_ were linked to favorable OS/LDMC and OS, respectively. While T_H_17 infiltration was associated with an improved prognosis of patients with HPV+ HNSCC (21), the current literature is balanced between studies reporting positive and negative impacts of T_reg_ infiltration on survival. Thereby, contradictory reports exist for both different HNSCC locations and for HPV-dependencies (41, 76, 77). Nevertheless, studies agreed on the relation of high CD8^+^ T cell infiltration and a positive impact on HNSCC patients’ survival (61, 78). Likewise, a higher frequency of CD8^+^ T lymphocytes as well as an elevated CD8:T_reg_ ratio correlated significantly with higher LRC and LDMC in our study. However, regarding the relation of CD8:T_reg_ ratio and survival properties of HNSCC patients, contradictory studies exist mentioning an adverse association (41, 79), no association or a favorable association (80, 81). A possible explanation for the inconclusive study situation was provided by Echarti et al. (82). In their investigation, the authors found a high T_reg_ infiltration to diminish OS in “immune desert” and “immune excluded” HNSCC, but a prolonged OS was seen in high T_reg_-infiltrated “inflamed” tumors. When assessing CD8^+^ subpopulations and their association with survival parameters of our cohort, cytotoxic GrzB^+^CD8^+^ T cells are unfavorable concerning clinical outcome for patients with Hypox15^high^ tumors, whereas for patients with Hypox15^low^ tumors the infiltration of active cytotoxic T cells seems to be linked to good survival. It is known that the hypoxic TME evades immune cell-mediated killing mechanisms by activating autophagy, forming autophagosomes, and neutralizing/degrading cytotoxic mediators, like GrzB (83). Additionally, whole tumor infiltration of PD-1^+^CD8^+^ T cells was associated with prolonged OS in the Hypox15^low^ subcohort and displaying a similar trend in the whole cohort, too. According to the literature, a high frequency of PD-1^+^CD8^+^ T cells was linked to favorable OS in patients with HPV- HNSCC, whereby the 25^th^ lowest percentile of these immune cell infiltrate pattern was used for grouping the patients (84).

When evaluating the clinical outcome of HNSCC patients according to p16 status in combination with immune cell infiltrate pattern of pDCs, cDC1, cDC2, CD3^+^, and CD8^+^ T cells, four subgroups can be classified: p16+/immune^high^, p16+/immune^low^, p16-/immune^high^, and p16-/immune^low^. In our investigation, especially patients with p16+ HNSCC and high immune cell infiltrate exhibited significantly better clinical outcomes in comparison to both p16- subgroups and tendentially prolonged survival compared to patients with p16+ HNSCC and low immune cell infiltrate. In literature, patients with HPV+ HNSCC and high infiltration of CD8^+^ T cells showed most favorable survival parameters (85) fitting our observations. Similarly, in terms of combined hypoxia/immune phenotype profiles different subgroups might be distinguished: hypoxia^low^/immune^high^, hypoxia^high^/immune^low^, hypoxia^low^/immune^low^, and hypoxia^high^/immune^high^. The last two phenotypes mentioned are also referred as a mixed or intermediate phenotype. In this regard, different investigators showed that the hypoxia^low^/immune^high^ phenotype was indicative of enhanced survival rates based on hypoxia- and immune response-related gene expression analysis of TCGA datasets or immunohistochemical staining of T cell (CD3) and hypoxia markers (carbonic anhydrase IX) (11, 86–88). In our study, we confirmed the positive association of the hypoxia^low^/immune^high^ phenotype with favorable clinical outcome of HNSCC patients. Though, different from the studies mentioned above, our phenotype clustering based on Hypox15 gene signature to determine the hypoxia status as well as on multiplex immunohistochemical staining combined with semi-automatic quantification of DCs and T cells at protein level to identify the immune cell infiltrate. In sum, favorable survival of patients with p16+ or Hypox15^low^ HNSCC seems to be particularly associated with a high infiltration of DCs and T cells. Thus, patients with p16+/immune^low^ and Hypox15^low^/immune^low^ HNSCC might benefit from therapeutically increased immune cell infiltrate.

Moreover, the infiltration of CLEC9A^+^ cDC1 and CD8^+^ T cells emerged as independent prognostic markers for improved clinical outcome besides clinicopathological parameters, like T stage, p16 status, and tumor localization, as shown in the multivariate Cox regression and our developed ICIS. By using a TCGA dataset of 497 HNSCC tissue samples, Liu et al. identified a significant association of CLEC9A gene and OS (univariate hazard ratio [HR]: 0.876, multivariate HR: 0.906) (89). Moreover, Furgiuele et al. reported CD8^+^ density to be an independent prognostic marker and an deduced ICIS, comprising tumor infiltration of T lymphocytes (CD8^+^, T_regs_) and macrophages (CD68^+^), improved HNSCC patients’ prognosis (90). Therefore, in the context of the still ongoing implementation of an ICIS for HNSCC (90, 91), cDC1 and CD8^+^ T cells are promising candidates for an ICIS to predict the clinical outcome of HNSCC patients.

## 5 Conclusion

In our study, we comprehensively investigated HNSCC infiltration patterns of several DC subtypes and T cell subpopulations, with respect to their phenotype and spatial distribution. The stromal tumor compartment is highly infiltrated by pDCs and T lymphocytes, with T_regs_ and exhausted CD8^+^ T cells being the predominant phenotypes and shaping an immunosuppressive tumor immune architecture. HPV-associated tumors showed significantly higher infiltration of several investigated immune cell populations, whereby tumors with hypoxia-associated gene signatures exhibited reduced infiltration. Furthermore, our study contributes to the identification of novel prognostic biomarkers for HNSCC, as especially cDC1 and CD8^+^ T cells were independent prognostic factors for clinical outcome and potentially contribute to the evaluation of a novel and more precise ICIS. These parameters may help to further stratify patient subgroups in need of treatment escalation. If confirmed in a validation cohort, combined treatment approaches with enhancing tumor perfusion (using hyperthermia, irradiation, potentially particle therapy) and immune checkpoint inhibitor therapy in high-risk tumors may be subject of future investigations. In patients with HNSCC receiving primary R(C)Tx, pretherapeutic diagnostic approaches with functional imaging such as ^18^F-fluoromisonidazol positron emission tomography (^18^F-FMISO PET) to detect hypoxic tumor areas and combining these findings with an ICIS based on the analysis of biopsy material could add prognostic information and lead to further individualized treatment strategies. This might improve the design of future radiochemo(immuno)therapy.

## Data availability statement

The original contributions presented in the study are included in the article/**Supplementary Material**. Further inquiries can be directed to the corresponding authors.

## Ethics statement

The studies involving humans were approved by Ethics Committee of Technische Universität Dresden. The studies were conducted in accordance with the local legislation and institutional requirements. The participants provided their written informed consent to participate in this study.

## Author contributions

JK: Conceptualization, Writing – original draft, Writing – review & editing, Data curation, Formal analysis, Investigation, Methodology, Resources. IP: Conceptualization, Data curation, Formal analysis, Investigation, Methodology, Writing – original draft, Writing – review & editing. RR: Conceptualization, Data curation, Formal analysis, Investigation, Methodology, Writing – original draft, Writing – review & editing, Visualization. AR: Conceptualization, Data curation, Formal analysis, Investigation, Methodology, Visualization, Writing – original draft, Writing – review & editing. SL: Data curation, Formal analysis, Investigation, Methodology, Visualization, Writing – original draft, Writing – review & editing. IB: Data curation, Formal analysis, Investigation, Methodology, Writing – original draft, Writing – review & editing. LR: Data curation, Formal analysis, Investigation, Methodology, Writing – original draft, Writing – review & editing. AL: Writing – original draft, Writing – review & editing, Funding acquisition, Resources. RW: Writing – original draft, Writing – review & editing, Funding acquisition. MK: Funding acquisition, Writing – original draft, Writing – review & editing, Resources. MS: Funding acquisition, Writing – original draft, Writing – review & editing, Conceptualization.

## References

[1] Sung H Ferlay J Siegel RL Laversanne M Soerjomataram I Jemal A. Global cancer statistics 2020: GLOBOCAN estimates of incidence and mortality worldwide for 36 cancers in 185 countries. CA Cancer J Clin. (2021) 71:209–49. doi: 10.3322/caac.21660 33538338

[2] Johnson DE Burtness B Leemans CR Lui VWY Bauman JE Grandis JR. Head and neck squamous cell carcinoma. Nat Rev Dis Primer. (2020) 6:92. doi: 10.1038/s41572-020-00224-3 PMC7944998 33243986

[3] Siegel RL Giaquinto AN Jemal A. Cancer statistics, 2024. CA Cancer J Clin. (2024) 74:12–49. doi: 10.3322/caac.21820 38230766

[4] Watermann C Pasternack H Idel C Ribbat-Idel J Braegelmann J Kuppler P. Recurrent HNSCC harbor an immunosuppressive tumor immune microenvironment suggesting successful tumor immune evasion. Clin Cancer Res. (2021) 27:632–44. doi: 10.1158/1078-0432.CCR-20-0197 33109740

[5] Mito I Takahashi H Kawabata-Iwakawa R Ida S Tada H Chikamatsu K. Comprehensive analysis of immune cell enrichment in the tumor microenvironment of head and neck squamous cell carcinoma. Sci Rep. (2021) 11:16134. doi: 10.1038/s41598-021-95718-9 34373557 PMC8352955

[6] Park JC Krishnakumar HN Saladi SV. Current and future biomarkers for immune checkpoint inhibitors in head and neck squamous cell carcinoma. Curr Oncol. (2022) 29:4185–98. doi: 10.3390/curroncol29060334 PMC9221564 35735443

[7] Wendt M Hammarstedt-Nordenvall L Zupancic M Friesland S Landin D Munck-Wikland E. Long-term survival and recurrence in oropharyngeal squamous cell carcinoma in relation to subsites, HPV, and p16-status. Cancers. (2021) 13:2553. doi: 10.3390/cancers13112553 34070952 PMC8196945

[8] Albers AE Qian X Kaufmann AM Coordes A. Meta analysis: HPV and p16 pattern determines survival in patients with HNSCC and identifies potential new biologic subtype. Sci Rep. (2017) 7:16715. doi: 10.1038/s41598-017-16918-w 29196639 PMC5711807

[9] Grønhøj C Jensen DH Dehlendorff C Marklund L Wagner S Mehanna H. Development and external validation of nomograms in oropharyngeal cancer patients with known HPV-DNA status: a European Multicentre Study (OroGrams). Br J Cancer. (2018) 118:1672–81. doi: 10.1038/s41416-018-0107-9 PMC6008433 29795309

[10] Linge A Löck S Gudziol V Nowak A Lohaus F von Neubeck C. Low cancer stem cell marker expression and low hypoxia identify good prognosis subgroups in HPV (–) HNSCC after postoperative radiochemotherapy: a multicenter study of the DKTK-ROG. Clin Cancer Res. (2016) 22:2639–49. doi: 10.1158/1078-0432.CCR-15-1990 26755529

[11] Wang H Zheng L. Construction of a hypoxia-derived gene model to predict the prognosis and therapeutic response of head and neck squamous cell carcinoma. Sci Rep. (2022) 12:13538. doi: 10.1038/s41598-022-17898-2 35945448 PMC9363468

[12] Li S-R Man Q-W Liu B. Development and validation of a novel hypoxia-related signature for prognostic and immunogenic evaluation in head and neck squamous cell carcinoma. Front Oncol. (2022) 12:943945. doi: 10.3389/fonc.2022.943945 36452497 PMC9702068

[13] Leemans CR Snijders PJ Brakenhoff RH. The molecular landscape of head and neck cancer. Nat Rev Cancer. (2018) 18:269–82. doi: 10.1038/nrc.2018.11 29497144

[14] Secrier M McGrath L Ng F Gulati S Raymond A Nuttall BR. Immune cell abundance and T-cell receptor landscapes suggest new patient stratification strategies in head and neck squamous cell carcinoma. Cancer Res Commun. (2023) 3:2133–45. doi: 10.1158/2767-9764.CRC-23-0155 PMC10588680 37819239

[15] Bruni D Angell HK Galon J. The immune contexture and Immunoscore in cancer prognosis and therapeutic efficacy. Nat Rev Cancer. (2020) 20:662–80. doi: 10.1038/s41568-020-0285-7 32753728

[16] Weed DT Zilio S McGee C Marnissi B Sargi Z Franzmann E. The tumor immune microenvironment architecture correlates with risk of recurrence in head and neck squamous cell carcinoma. Cancer Res. (2023) 83:3886–900. doi: 10.1158/0008-5472.CAN-23-0379 PMC10690086 37602821

[17] Hadler-Olsen E Wirsing AM. Tissue-infiltrating immune cells as prognostic markers in oral squamous cell carcinoma: a systematic review and meta-analysis. Br J Cancer. (2019) 120:714–27. doi: 10.1038/s41416-019-0409-6 PMC6461751 30808992

[18] Wculek SK Cueto FJ Mujal AM Melero I Krummel MF Sancho D. Dendritic cells in cancer immunology and immunotherapy. Nat Rev Immunol. (2020) 20:7–24. doi: 10.1038/s41577-019-0210-z 31467405

[19] Böttcher JP Reis e Sousa C. The role of type 1 conventional dendritic cells in cancer immunity. Trends Cancer. (2018) 4:784–92. doi: 10.1016/j.trecan.2018.09.001 PMC6207145 30352680

[20] Michea P Noël F Zakine E Czerwinska U Sirven P Abouzid O. Adjustment of dendritic cells to the breast-cancer microenvironment is subset specific. Nat Immunol. (2018) 19:885–97. doi: 10.1038/s41590-018-0145-8 30013147

[21] Minohara K Imai M Matoba T Wing JB Shime H Odanaka M. Mature dendritic cells enriched in regulatory molecules may control regulatory T cells and the prognosis of head and neck cancer. Cancer Sci. (2023) 114:1256. doi: 10.1111/cas.15698 36529525 PMC10067395

[22] Binnewies M Roberts EW Kersten K Chan V Fearon DF Merad M. Understanding the tumor immune microenvironment (TIME) for effective therapy. Nat Med. (2018) 24:541–50. doi: 10.1038/s41591-018-0014-x PMC5998822 29686425

[23] Kießler M Plesca I Sommer U Wehner R Wilczkowski F Müller L. Tumor-infiltrating plasmacytoid dendritic cells are associated with survival in human colon cancer. J Immunother Cancer. (2021) 9:e001813. doi: 10.1136/jitc-2020-001813 33762320 PMC7993360

[24] Balermpas P Rödel F Krause M Linge A Lohaus F Baumann M. The PD-1/PD-L1 axis and human papilloma virus in patients with head and neck cancer after adjuvant chemoradiotherapy: a multicentre study of the German Cancer Consortium Radiation Oncology Group (DKTK-ROG). Int J Cancer. (2017) 141:594–603. doi: 10.1002/ijc.30770 28480996

[25] Havel JJ Chowell D Chan TA. The evolving landscape of biomarkers for checkpoint inhibitor immunotherapy. Nat Rev Cancer. (2019) 19:133–50. doi: 10.1038/s41568-019-0116-x PMC6705396 30755690

[26] Zhang S Zhang W Zhang J. Comprehensive analysis of immune cell infiltration and significant genes in head and neck squamous cell carcinoma. Oral Oncol. (2022) 126:105755. doi: 10.1016/j.oraloncology.2022.105755 35144208

[27] Knebel M Körner S Kühn JP Wemmert S Brust L Smola S. Prognostic impact of intra-and peritumoral immune cell subpopulations in head and neck squamous cell carcinomas–comprehensive analysis of the TCGA-HNSC cohort and immunohistochemical validation on 101 patients. Front Immunol. (2023) 14:1172768. doi: 10.3389/fimmu.2023.1172768 37383237 PMC10294051

[28] Rühle A Todorovic J Spohn SS Gkika E Becker C Knopf A. Prognostic value of tumor-infiltrating immune cells and immune checkpoints in elderly head-and-neck squamous cell carcinoma patients undergoing definitive (chemo) radiotherapy. Radiat Oncol. (2022) 17:1–12. doi: 10.1186/s13014-022-02153-9 36376922 PMC9661751

[29] Ding Y Chu L Cao Q Lei H Li X Zhuang Q. A meta-validated immune infiltration-related gene model predicts prognosis and immunotherapy sensitivity in HNSCC. BMC Cancer. (2023) 23:1–18. doi: 10.1186/s12885-023-10532-y 36639648 PMC9837972

[30] Linge A Schötz U Löck S Lohaus F von Neubeck C Gudziol V. Comparison of detection methods for HPV status as a prognostic marker for loco-regional control after radiochemotherapy in patients with HNSCC. Radiother Oncol. (2018) 127:27–35. doi: 10.1016/j.radonc.2017.12.007 29295747

[31] Toustrup K Sørensen BS Nordsmark M Busk M Wiuf C Alsner J. Development of a hypoxia gene expression classifier with predictive impact for hypoxic modification of radiotherapy in head and neck cancer. Cancer Res. (2011) 71:5923–31. doi: 10.1158/0008-5472.CAN-11-1182 21846821

[32] Rupp L Resag A Potkrajcic V Warm V Wehner R Jöhrens K. Prognostic impact of the post-treatment T cell composition and spatial organization in soft tissue sarcoma patients treated with neoadjuvant hyperthermic radio (chemo) therapy. Front Immunol. (2023) 14:1185197. doi: 10.3389/fimmu.2023.1185197 37261361 PMC10228739

[33] Bayerl F Bejarano DA Bertacchi G Doffin A Gobbini E Hubert M. Guidelines for visualization and analysis of DC in tissues using multiparameter fluorescence microscopy imaging methods. Eur J Immunol. (2023) 53:2249923. doi: 10.1002/eji.202249923 36623939

[34] R Core Team. R: a language and environment for statistical computing (2023). Available online at: https://www.R-project.org/ (Accessed March 25, 2024).

[35] Johnson KS. phenoptr: inForm Helper Functions (2022). Available online at: https://akoyabio.github.io/phenoptr/ (Accessed March 25, 2024).

[36] Johnson KS. phenoptrReports: Create reports using Phenoptics data (2022). Available online at: https://akoyabio.github.io/phenoptrReports/ https://github.com/akoyabio/phenoptrReports/ (Accessed March 25, 2024).

[37] Schindelin J Arganda-Carreras I Frise E Kaynig V Longair M Pietzsch T. Fiji: an open-source platform for biological-image analysis. Nat Methods. (2012) 9:676–82. doi: 10.1038/nmeth.2019 PMC3855844 22743772

[38] ten Cate V. Forplo: flexible forest plots (2023). Available online at: https://CRAN.R-project.org/package=forplo (Accessed March 25, 2024).

[39] Ribbat-Idel J Perner S Kuppler P Klapper L Krupar R Watermann C. Immunologic “cold” squamous cell carcinomas of the head and neck are associated with an unfavorable prognosis. Front Med. (2021) 8:622330. doi: 10.3389/fmed.2021.622330 PMC7873597 33585526

[40] Galon J Bruni D. Tumor immunology and tumor evolution: intertwined histories. Immunity. (2020) 52:55–81. doi: 10.1016/j.immuni.2019.12.018 31940273

[41] Wondergem NE Nauta IH Muijlwijk T Leemans CR van de Ven R. The immune microenvironment in head and neck squamous cell carcinoma: on subsets and subsites. Curr Oncol Rep. (2020) 22:1–14. doi: 10.1007/s11912-020-00938-3 32602047 PMC7324425

[42] Plesca I Benešová I Beer C Sommer U Müller L Wehner R. Clinical significance of tumor-infiltrating conventional and plasmacytoid dendritic cells in pancreatic ductal adenocarcinoma. Cancers. (2022) 14:1216. doi: 10.3390/cancers14051216 35267524 PMC8909898

[43] Fan C Wu J Shen Y Hu H Wang Q Mao Y. Hypoxia promotes the tolerogenic phenotype of plasmacytoid dendritic cells in head and neck squamous cell carcinoma. Cancer Med. (2022) 11:922–30. doi: 10.1002/cam4.4511 PMC8855917 34964283

[44] Koucký V Hladíková K Táborská E Bouček J Grega M Špíšek R. The cytokine milieu compromises functional capacity of tumor-infiltrating plasmacytoid dendritic cells in HPV-negative but not in HPV-positive HNSCC. Cancer Immunol Immunother. (2021) 70:2545–57. doi: 10.1007/s00262-021-02874-y PMC10992977 33569630

[45] Idel C Ribbat-Idel J Klapper L Krupar R Bruchhage K-L Dreyer E. Spatial distribution of immune cells in head and neck squamous cell carcinomas. Front Oncol. (2021) 11:712788. doi: 10.3389/fonc.2021.712788 34778030 PMC8581660

[46] Ralli M Grasso M Gilardi A Ceccanti M Messina M Tirassa P. The role of cytokines in head and neck squamous cell carcinoma: A review. Clin Ter. (2020) 171:268–74. doi: 10.7417/CT.2020.2225 32323717

[47] Qureshi HA Zhu X Yang GH Steadele M Pierce RH Futran ND. Impact of HPV status on immune responses in head and neck squamous cell carcinoma. Oral Oncol. (2022) 127:105774. doi: 10.1016/j.oraloncology.2022.105774 35219073

[48] Lu H Dai W Guo J Wang D Wen S Yang L. High abundance of intratumoral γδ T cells favors a better prognosis in head and neck squamous cell carcinoma: a bioinformatic analysis. Front Immunol. (2020) 11:573920. doi: 10.3389/fimmu.2020.573920 33101298 PMC7555127

[49] Lee M-H Chang JT-C Liao C-T Chen Y-S Kuo M-L Shen C-R. Interleukin 17 and peripheral IL-17-expressing T cells are negatively correlated with the overall survival of head and neck cancer patients. Oncotarget. (2018) 9:9825. doi: 10.18632/oncotarget.v9i11 29515773 PMC5839404

[50] Norouzian M Mehdipour F Ashraf MJ Khademi B Ghaderi A. Regulatory and effector T cell subsets in tumor-draining lymph nodes of patients with squamous cell carcinoma of head and neck. BMC Immunol. (2022) 23:56. doi: 10.1186/s12865-022-00530-3 36376825 PMC9664675

[51] Agerholm R Bekiaris V. Evolved to protect, designed to destroy: IL-17-producing γδ T cells in infection, inflammation, and cancer. Eur J Immunol. (2021) 51:2164–77. doi: 10.1002/eji.202049119 34224140

[52] Xu L Jin Y Qin X. Comprehensive analysis of significant genes and immune cell infiltration in HPV-related head and neck squamous cell carcinoma. Int Immunopharmacol. (2020) 87:106844. doi: 10.1016/j.intimp.2020.106844 32738592

[53] Pokrývková B Grega M Klozar J Vencálek O Nunvář J Tachezy R. PD1+ CD8+ Cells are an independent prognostic marker in patients with head and neck cancer. Biomedicines. (2022) 10:2794. doi: 10.3390/biomedicines10112794 36359314 PMC9687997

[54] Boucek J Mrkvan T Chovanec M Kuchar M Betka J Boucek V. Regulatory T cells and their prognostic value for patients with squamous cell carcinoma of the head and neck. J Cell Mol Med. (2010) 14:426–33. doi: 10.1111/j.1582-4934.2008.00650.x PMC3837595 19183242

[55] Veigas F Mahmoud YD Merlo J Rinflerch A Rabinovich GA Girotti MR. Immune checkpoints pathways in head and neck squamous cell carcinoma. Cancers. (2021) 13:1018. doi: 10.3390/cancers13051018 33804419 PMC7957692

[56] Lydiatt WM Patel SG O’Sullivan B Brandwein MS Ridge JA Migliacci JC. Head and neck cancers—major changes in the American Joint Committee on cancer eighth edition cancer staging manual. CA Cancer J Clin. (2017) 67:122–37. doi: 10.3322/caac.21389 28128848

[57] Zhu G Amin N Herberg ME Maroun CA Wang H Guller M. Association of tumor site with the prognosis and immunogenomic landscape of human papillomavirus–related head and neck and cervical cancers. JAMA Otolaryngol Neck Surg. (2022) 148:70–9. doi: 10.1001/jamaoto.2021.3228 PMC8603246 34792560

[58] Conarty JP Wieland A. The tumor-specific immune landscape in HPV+ Head and neck cancer. Viruses. (2023) 15:1296. doi: 10.3390/v15061296 37376596 PMC10301020

[59] Partlová S Bouček J Kloudová K Lukešová E Zábrodský M Grega M. Distinct patterns of intratumoral immune cell infiltrates in patients with HPV-associated compared to non-virally induced head and neck squamous cell carcinoma. Oncoimmunology. (2015) 4:e965570. doi: 10.4161/21624011.2014.965570 25949860 PMC4368144

[60] Gameiro SF Ghasemi F Barrett JW Koropatnick J Nichols AC Mymryk JS. Treatment-naïve HPV+ head and neck cancers display a T-cell-inflamed phenotype distinct from their HPV-counterparts that has implications for immunotherapy. Oncoimmunology. (2018) 7:e1498439. doi: 10.1080/2162402X.2018.1498439 30288365 PMC6169583

[61] Lechien JR Seminerio I Descamps G Mat Q Mouawad F Hans S. Impact of HPV infection on the immune system in oropharyngeal and non-oropharyngeal squamous cell carcinoma: a systematic review. Cells. (2019) 8:1061. doi: 10.3390/cells8091061 31510065 PMC6769551

[62] Cheng D Qiu K Rao Y Mao M Li L Wang Y. Proliferative exhausted CD8+ T cells exacerbate long-lasting anti-tumor effects in human papillomavirus-positive head and neck squamous cell carcinoma. Elife. (2023) 12:e82705. doi: 10.7554/eLife.82705 36811599 PMC9946444

[63] Kansy BA Wehrs TP Bruderek K Si Y Ludwig S Droege F. HPV-associated head and neck cancer is characterized by distinct profiles of CD8+ T cells and myeloid-derived suppressor cells. Cancer Immunol Immunother. (2023) 72:4367–83. doi: 10.1007/s00262-023-03571-8 PMC10700222 38019346

[64] Taylor CT Colgan SP. Regulation of immunity and inflammation by hypoxia in immunological niches. Nat Rev Immunol. (2017) 17:774–85. doi: 10.1038/nri.2017.103 PMC5799081 28972206

[65] Telarovic I Wenger RH Pruschy M. Interfering with tumor hypoxia for radiotherapy optimization. J Exp Clin Cancer Res. (2021) 40:1–26. doi: 10.1186/s13046-021-02000-x 34154610 PMC8215813

[66] Kim HAJ Zeng PY Shaikh MH Mundi N Ghasemi F Di Gravio E. All HPV-negative head and neck cancers are not the same: Analysis of the TCGA dataset reveals that anatomical sites have distinct mutation, transcriptome, hypoxia, and tumor microenvironment profiles. Oral Oncol. (2021) 116:105260. doi: 10.1016/j.oraloncology.2021.105260 33725617

[67] Peng C Ye H Li Z Duan X Yang W Yi Z. Multi-omics characterization of a scoring system to quantify hypoxia patterns in patients with head and neck squamous cell carcinoma. J Transl Med. (2023) 21:15. doi: 10.1186/s12967-022-03869-8 36627705 PMC9830846

[68] Koukourakis IM Gkegka AG Xanthopoulou E Nanos C Giatromanolaki A Koukourakis MI. Prognostic and predictive relevance of tumor-infiltrating lymphocytes in squamous cell head–neck cancer patients treated with radical Radiotherapy/Chemo-radiotherapy. Curr Oncol. (2022) 29:4274–84. doi: 10.3390/curroncol29060342 PMC9222114 35735451

[69] Sureshbabu SK Godbole JH Vaibhaw A Chiplunkar SV. Immunosuppressive microenvironment in oral cancer: implications for cancer immunotherapy. Explor Immunol. (2021) 1:166–98. doi: 10.37349/ei.2021.00013

[70] Han N Li X Wang Y Wang L Zhang C Zhang Z. Increased tumor-infiltrating plasmacytoid dendritic cells promote cancer cell proliferation and invasion via TNF-α/NF-κB/CXCR-4 pathway in oral squamous cell carcinoma. J Cancer. (2021) 12:3045–56. doi: 10.7150/jca.55580 PMC8040884 33854604

[71] Yang L-L Mao L Wu H Chen L Deng W-W Xiao Y. pDC depletion induced by CD317 blockade drives the antitumor immune response in head and neck squamous cell carcinoma. Oral Oncol. (2019) 96:131–9. doi: 10.1016/j.oraloncology.2019.07.019 31422204

[72] Meiser P Knolle MA Hirschberger A de Almeida GP Bayerl F Lacher S. A distinct stimulatory cDC1 subpopulation amplifies CD8+ T cell responses in tumors for protective anti-cancer immunity. Cancer Cell. (2023) 41:1498–515. doi: 10.1016/j.ccell.2023.06.008 37451271

[73] Santegoets SJ Duurland CL Jordanova EJ van Ham VJ Ehsan I Loof NM. CD163+ cytokine-producing cDC2 stimulate intratumoral type 1 T cell responses in HPV16-induced oropharyngeal cancer. J Immunother Cancer. (2020) 8:e001053. doi: 10.1136/jitc-2020-001053 32771994 PMC7418847

[74] Hoffmann C Noel F Grandclaudon M Massenet-Regad L Michea P Sirven P. PD-L1 and ICOSL discriminate human secretory and helper dendritic cells in cancer, allergy and autoimmunity. Nat Commun. (2022) 13:1983. doi: 10.1038/s41467-022-29516-w 35418195 PMC9008048

[75] Nguyen N Bellile E Thomas D McHugh J Rozek L Virani S. Tumor infiltrating lymphocytes and survival in patients with head and neck squamous cell carcinoma. Head Neck. (2016) 38:1074–84. doi: 10.1002/hed.24406 PMC4900934 26879675

[76] Fialová A Koucký V Hajdušková M Hladíková K Špíšek R. Immunological network in head and neck squamous cell carcinoma—a prognostic tool beyond HPV status. Front Oncol. (2020) 10:1701. doi: 10.3389/fonc.2020.01701 33042814 PMC7522596

[77] Rad HS Shiravand Y Radfar P Ladwa R Perry C Han X. Understanding the tumor microenvironment in head and neck squamous cell carcinoma. Clin Transl Immunol. (2022) 11:e1397. doi: 10.1002/cti2.1397 PMC9170522 35686027

[78] Borsetto D Tomasoni M Payne K Polesel J Deganello A Bossi P. Prognostic significance of CD4+ and CD8+ tumor-infiltrating lymphocytes in head and neck squamous cell carcinoma: a meta-analysis. Cancers. (2021) 13:781. doi: 10.3390/cancers13040781 33668519 PMC7918220

[79] Lequerica-Fernández P Suárez-Canto J Rodriguez-Santamarta T Rodrigo JP Suárez-Sánchez FJ Blanco-Lorenzo V. Prognostic relevance of CD4+, CD8+ and FOXP3+ TILs in oral squamous cell carcinoma and correlations with PD-L1 and cancer stem cell markers. Biomedicines. (2021) 9:653. doi: 10.3390/biomedicines9060653 34201050 PMC8227658

[80] Sanchez-Canteli M Granda-Díaz R del Rio-Ibisate N Allonca E López-Alvarez F Agorreta J. PD-L1 expression correlates with tumor-infiltrating lymphocytes and better prognosis in patients with HPV-negative head and neck squamous cell carcinomas. Cancer Immunol Immunother. (2020) 69:2089–100. doi: 10.1007/s00262-020-02604-w PMC11027666 32448984

[81] Ni Y-H Zhang X Lu Z Huang X-F Wang Z-Y Yang Y. Tumor-infiltrating CD1a+ DCs and CD8+/FoxP3+ ratios served as predictors for clinical outcomes in tongue squamous cell carcinoma patients. Pathol Oncol Res. (2020) 26:1687–95. doi: 10.1007/s12253-019-00701-5 31606786

[82] Echarti A Hecht M Büttner-Herold M Haderlein M Hartmann A Fietkau R. CD8+ and regulatory T cells differentiate tumor immune phenotypes and predict survival in locally advanced head and neck cancer. Cancers. (2019) 11:1398. doi: 10.3390/cancers11091398 31546872 PMC6769847

[83] Abou Khouzam R Janji B Thiery J Zaarour RF Chamseddine AN Mayr H. Hypoxia as a potential inducer of immune tolerance, tumor plasticity and a driver of tumor mutational burden: Impact on cancer immunotherapy. Semin Cancer Biol. (2023) 97:104–23. doi: 10.1016/j.semcancer.2023.11.008 38029865

[84] Muijlwijk T Nijenhuis DN Ganzevles SH Brink A Ke C Fass JN. Comparative analysis of immune infiltrates in head and neck cancers across anatomical sites. J Immunother Cancer. (2024) 12:e007573. doi: 10.1136/jitc-2023-007573 38212122 PMC10806653

[85] Zhu Y Zhu X Diao W Liang Z Gao Z Chen X. Correlation of immune makers with HPV 16 infections and the prognosis in oropharyngeal squamous cell carcinoma. Clin Oral Investig. (2023) 27:1423–33. doi: 10.1007/s00784-023-04926-2 PMC10102146 36884083

[86] Brooks JM Menezes AN Ibrahim M Archer L Lal N Bagnall CJ. Development and validation of a combined hypoxia and immune prognostic classifier for head and neck cancer. Clin Cancer Res. (2019) 25:5315–28. doi: 10.1158/1078-0432.CCR-18-3314 31182433

[87] Rühle A Grosu A-L Wiedenmann N Stoian R Haehl E Zamboglou C. Immunohistochemistry-based hypoxia-immune prognostic classifier for head-and-neck cancer patients undergoing chemoradiation–Post-hoc analysis from a prospective imaging trial. Radiother Oncol. (2021) 159:75–81. doi: 10.1016/j.radonc.2021.03.014 33753155

[88] Zhu G Yang K Xu C Feng R Li W Ma J. Development of a prediction model for radiotherapy response among patients with head and neck squamous cell carcinoma based on the tumor immune microenvironment and hypoxia signature. Cancer Med. (2022) 11:4673–87. doi: 10.1002/cam4.4791 PMC9741991 35505641

[89] Liu G Zeng X Wu B Zhao J Pan Y. RNA-Seq analysis of peripheral blood mononuclear cells reveals unique transcriptional signatures associated with radiotherapy response of nasopharyngeal carcinoma and prognosis of head and neck cancer. Cancer Biol Ther. (2020) 21:139–46. doi: 10.1080/15384047.2019.1670521 PMC7012055 31698994

[90] Furgiuele S Descamps G Lechien JR Dequanter D Journe F Saussez S. Immunoscore combining CD8, foxP3, and CD68-positive cells density and distribution predicts the prognosis of head and neck cancer patients. Cells. (2022) 11:2050. doi: 10.3390/cells11132050 35805132 PMC9266282

[91] Zamani R Rezaei N. Immune-scoring in head and neck squamous cell carcinoma: a scoping review. Expert Rev Clin Immunol. (2023), 1–9. doi: 10.1080/1744666X.2023.2262140. Epub ahead of print. 37750738

