# Supplementary material for: Type I conventional dendritic cells and CD8+ T cells predict favorable clinical outcome of head and neck squamous cell carcinoma patients
Source: Front Immunol. 2024 Jun 13;15:1414298. doi: 10.3389/fimmu.2024.1414298 (PMC11208331; doi:10.3389/fimmu.2024.1414298)
Supplement: Supplementary file 2 [file Table_2.docx]

Supplementary Material

Type I conventional dendritic cells and CD8^+^ T cells predict favorable clinical outcome of head and neck squamous cell carcinoma patients

Johanna Kirchner, Ioana Plesca, Rebecca Rothe, Antonia Resag, Steffen Löck, Iva Benešová, Luise Rupp, Annett Linge, Rebekka Wehner, Mechthild Krause*, Marc Schmitz*

*** Correspondence:**

Corresponding Authors: [marc.schmitz@tu-dresden.de](mailto:marc.schmitz@tu-dresden.de), [mechthild.krause@uniklinikum-dresden.de](mailto:mechthild.krause@uniklinikum-dresden.de)

# Supplementary Tables

**Supplementary Table 1: Patient characteristics of the whole HNSCC cohort**

| **whole cohort (n = 56)** | | **n** | **%** |
| --- | --- | --- | --- |
| **Age**  (years) | median  range | 57.4  45.3 – 77.8 |  |
| **Gender** | male  female | 47  9 | 83.9  16.1 |
| **Grade** | G2  G3 | 20  36 | 35.7  64.3 |
| **p16** | negative  positive | 32  24 | 57.1  42.9 |
| **Hypoxia** | Hypox15 low  Hypox15 high | 36  20 | 64.3  35.7 |
| **Location** | oropharynx  oral cavity | 43  13 | 76.8  23.2 |
| **TNM** | T1-T2  T3-T4  N0  N1-N3 | 42  14  26  30 | 75.0  25.0  46.4  53.6 |
| **Smoking** | 0  1  2 | 9  28  19 | 16.1  50.0  33.9 |
| **Alcohol** | 0  1  2 | 13  12  31 | 23.2  21.4  55.4 |
| **OS**  (months) | median  range | 62.1  7.4-167.7 |  |
| **LRC**  (months) | median  range | 61.0  3.0-167.7 |  |
| **LDMC**  (months) | median  range | 60.8  3.0-167.7 |  |

**Supplementary Table 2: Multiplex IHC staining panels for DCs, CD8^+^ T cells and T_H_ cells.**

| **Antigen** | **Antibody dilution** | **Supplier** | **Catalog nr.**  **(Clone)** | **Species** | **Fluorophore** | **Fluorophore dilution** |
| --- | --- | --- | --- | --- | --- | --- |
| **DC Panel** | | | | | | |
| BDCA-2 | 1:75 | R&D Systems | AF1376  (polyclonal) | Goat | 690 | 1:75 |
| IRF7 | 1:75 | Santa Cruz | sc-74472  (G-8) | Mouse | 570 | 1:75 |
| CLEC9A | 1:175 | Abcam | ab223188  (EPR22324) | Rabbit | 650 | 1:350 |
| CLEC10A | 1:100 | Human Protein Atlas | HPA021937  (polyclonal) | Rabbit | 520 | 1:75 |
| CD1c | 1:50 | Novusbio | NBP2-46123  (2FA) | Mouse | 620 | 1:150 |
| PanCK | - | Roche (Ventana) | 5267145001 (AE1/AE3/PCK26) | Mouse | 540 | 1:400 |
| **CD8 Panel** | | | | | | |
| CD8 | - | Roche (Ventana) | 5937248001  (SP57) | Rabbit | 520 | 1:75 |
| PD-1 | - | Roche (Ventana) | 7099029001  (NAT105) | Mouse | 620 | 1:100 |
| LAG-3 | 1:50 | Cell Signaling Technology | #15372  (D2G4O) | Rabbit | 540 | 1:50 |
| GrzB | 1:75 | Dako | M7235  (Grb-7) | Mouse | 570 | 1:100 |
| Ki67 | 1:100 | Dako | M7240  (MIB-1) | Mouse | 690 | 1:150 |
| PanCK | - | Roche (Ventana) | 5267145001 (AE1/AE3/PCK26) | Mouse | 650 | 1:400 |
| **T_H_ panel** | | | | | | |
| CD3 | - | Roche (Ventana) | 5278422001  (2GV6) | Rabbit | 540 | 1:250 |
| FoxP3 | 1:50 | Abcam | ab20034  (236A/E7) | Mouse | 520 | 1:50 |
| T-bet | 1:75 | Cell Signaling Technology | #13232  (D6N8B) | Rabbit | 650 | 1:75 |
| GATA3 | 1:75 | Cell Signaling Technology | #5852  (D13C9) | Rabbit | 570 | 1:100 |
| RORγT | 1:100 | Merck Millipore | MABF81  (6F3.1) | Mouse | 620 | 1:125 |
| PanCK | - | Roche (Ventana) | 5267145001 (AE1/AE3/PCK26) | Mouse | 690 | 1:75 |

**Supplementary Table 3: Patient characteristics of p16+- and p16- subcohorts.**

| **p16+ subcohort (n = 24)** | | **n** | **%** | **p16- subcohort (n = 32)** | | **n** | **%** |
| --- | --- | --- | --- | --- | --- | --- | --- |
| **Age**  (years) | median  range | 59.9  46.5 – 74.7 |  | **Age**  (years) | median  range | 55.2  45.3 – 77.8 |  |
| **Gender** | male  female | 19  5 | 79.2  20.8 | **Gender** | male  female | 28  4 | 87.5  12.5 |
| **Grade** | G2  G3 | 7  17 | 29.2  70.8 | **Grade** | G2  G3 | 13  19 | 40.6  59.4 |
| **p16** | negative  positive | 0  24 | 0  100 | **p16** | negative  positive | 32  0 | 100  0 |
| **Hypoxia** | Hypox15 low  Hypox15 high | 19  5 | 79.2  20.8 | **Hypoxia** | Hypox15 low  Hypox15 high | 17  15 | 53.1  46.9 |
| **Location** | oropharynx  oral cavity | 24  0 | 100  0 | **Location** | oropharynx  oral cavity | 19  13 | 59.4  40.6 |
| **TNM** | T1-T2  T3  N0  N1-N3 | 22  2  7  17 | 91.7  8.3  29.2  70.8 | **TNM** | T1-T2  T3-T4  N0  N1-N2 | 20  12  19  13 | 62.5  37.5  59.4  40.6 |
| **Smoking** | 0  1  2 | 8  15  1 | 33.3  62.5  4.2 | **Smoking** | 0  1  2 | 1  13  18 | 3.1  40.6  56.3 |
| **Alcohol** | 0  1  2 | 10  7  7 | 41.2  29.2  29.2 | **Alcohol** | 0  1  2 | 3  5  24 | 9.4  15.6  75.0 |
| **OS**  (months) | median  range | 65.7  16.1-167.7 |  | **OS**  (months) | median  range | 58.6  7.4-136.9 |  |
| **LRC**  (months) | median  range | 64.2  5.3-167.7 |  | **LRC**  (months) | median  range | 58.6  3.0-136.9 |  |
| **LDMC**  (months) | median  range | 64.2  5.3-167.7 |  | **LDMC**  (months) | median  range | 56.1  3.0-136.9 |  |

**Supplementary Table 4: Patient characteristics of Hypox15 high- and Hypox15 low- subcohorts.**

| **Hypox15 high subcohort (n = 20)** | | **n** | **%** | **Hypox15 low subcohort**  **(n = 36)** | | **n** | **%** |
| --- | --- | --- | --- | --- | --- | --- | --- |
| **Age**  (years) | median  range | 54.5  45.3 – 76.0 |  | **Age**  (years) | median  range | 58.2  46.7 – 77.8 |  |
| **Gender** | male  female | 18  2 | 90.0  10.0 | **Gender** | male  female | 29  7 | 80.6  19.4 |
| **Grade** | G2  G3 | 11  9 | 55.0  45.0 | **Grade** | G2  G3 | 9  27 | 25.0  75.0 |
| **p16** | negative  positive | 15  5 | 75.0  25.0 | **p16** | negative  positive | 17  19 | 47.2  52.8 |
| **Hypoxia** | Hypox15 low  Hypox15 high | 0  20 | 0  100 | **Hypoxia** | Hypox15 low  Hypox15 high | 36  0 | 100  0 |
| **Location** | oropharynx  oral cavity | 11  9 | 55.0  45.0 | **Location** | oropharynx  oral cavity | 32  4 | 88.9  11.1 |
| **TNM** | T2  T3-T4  N0  N1-N2 | 13  7  12  8 | 65.0  35.0  60.0  40.0 | **TNM** | T1-T2  T3  N0  N1-N3 | 29  7  14  22 | 80.6  19.4  38.9  61.1 |
| **Smoking** | 0  1  2 | 2  10  8 | 10.0  50.0  40.0 | **Smoking** | 0  1  2 | 7  18  11 | 19.4  50.0  30.6 |
| **Alcohol** | 0  1  2 | 4  4  12 | 20.0  20.0  60.0 | **Alcohol** | 0  1  2 | 9  8  19 | 25.0  22.2  52.8 |
| **OS**  (months) | median  range | 53.9  7.4-136.9 |  | **OS**  (months) | median  range | 66.3  15.0-167.7 |  |
| **LRC**  (months) | median  range | 53.6  3.7-136.9 |  | **LRC**  (months) | median  range | 65.6  3.0-167.7 |  |
| **LDMC**  (months) | median  range | 42.9  3.7-136.9 |  | **LDMC**  (months) | median  range | 65.6  3.0-167.7 |  |

# Supplementary Figures

**
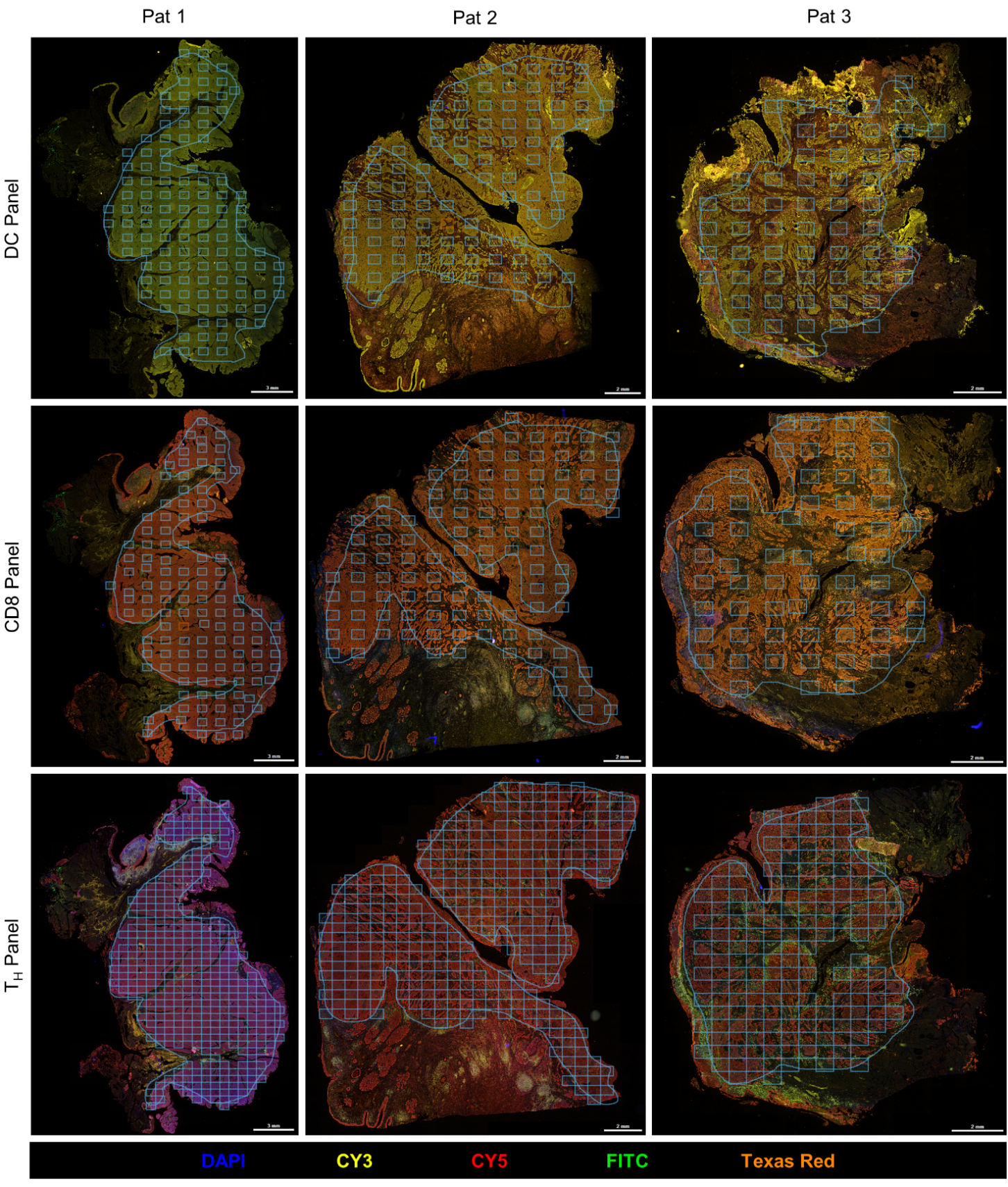
**

**Supplementary Figure 1:** **Representative overview scans of whole HNSCC tissue sections of three patients stained with DC-, CD8-, and T_H_-panel.** Overview scans (100x magnification, five fluorescence channels) were acquired to define the position of multispectral images used for subsequent data analysis (200x magnification, cyan rectangles) in the annotated whole tumor area (WTA, cyan line).

**
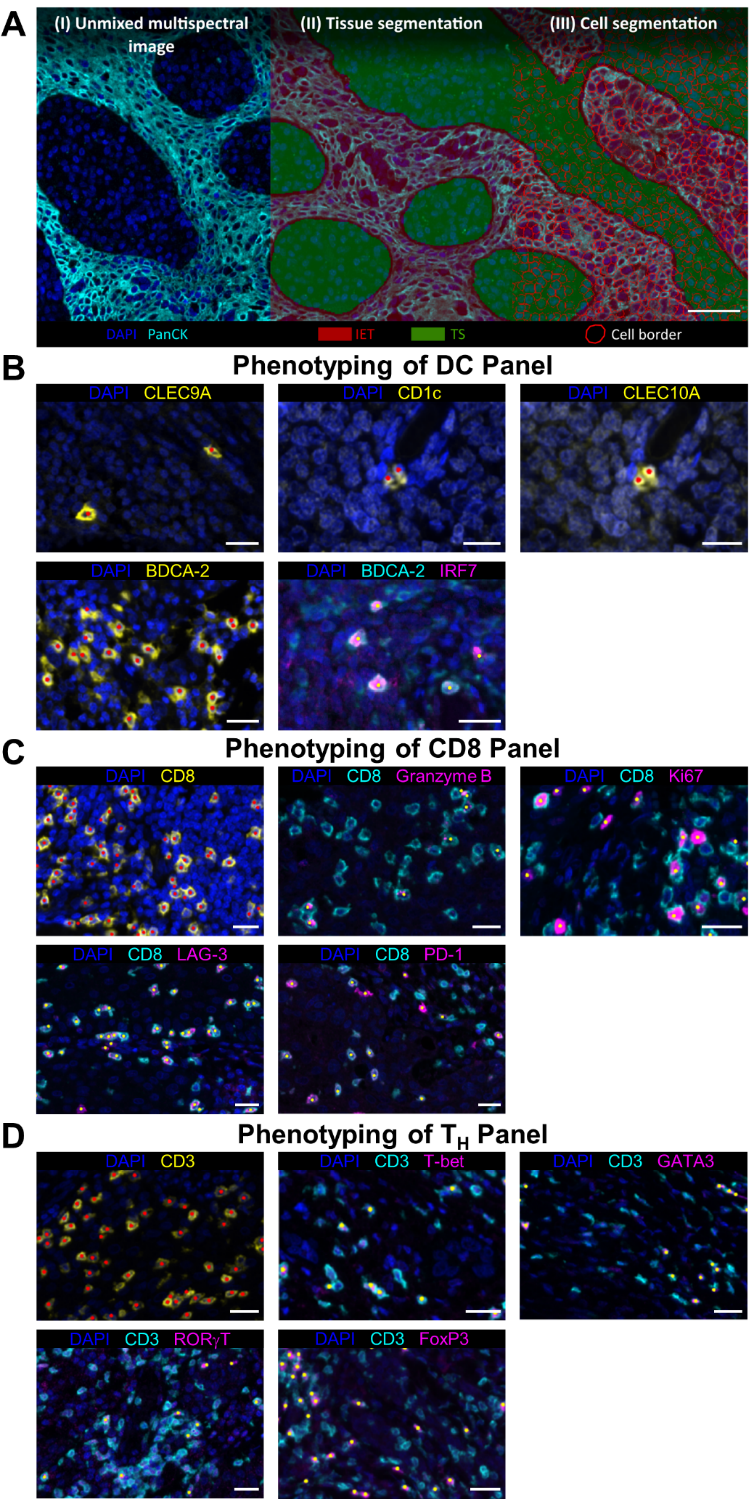
**

**Supplementary Figure 2:** **Image analysis based on a semi-automatic approach. A)** Algorithms were trained to segment spectrally unmixed multispectral images. Tissue segmentation includes the differentiation between intraepithelial PanCK+ tumor tissue (IET, red) from stromal tissue (TS, green) and cell segmentation identifies single cells based on DAPI staining (scale: 50 µm). Immune cells were phenotyped based on the signal intensity of the respective marker stained by **B)** DC panel, **C)** CD8 panel, or **D)** T_H_ panel (scale: 25 µm). Positive cells are marked with a red (CLEC9A, CD1c, CLEC10A, BDCA-2, CD8, CD3) or yellow dot (IRF7, GrzB, Ki67, LAG-3, PD-1, T-bet, GATA3, RORγT, FoxP3). Markers depicted in cyan (BDCA-2, CD8, CD3) are shown for better perceptibility of the cells.


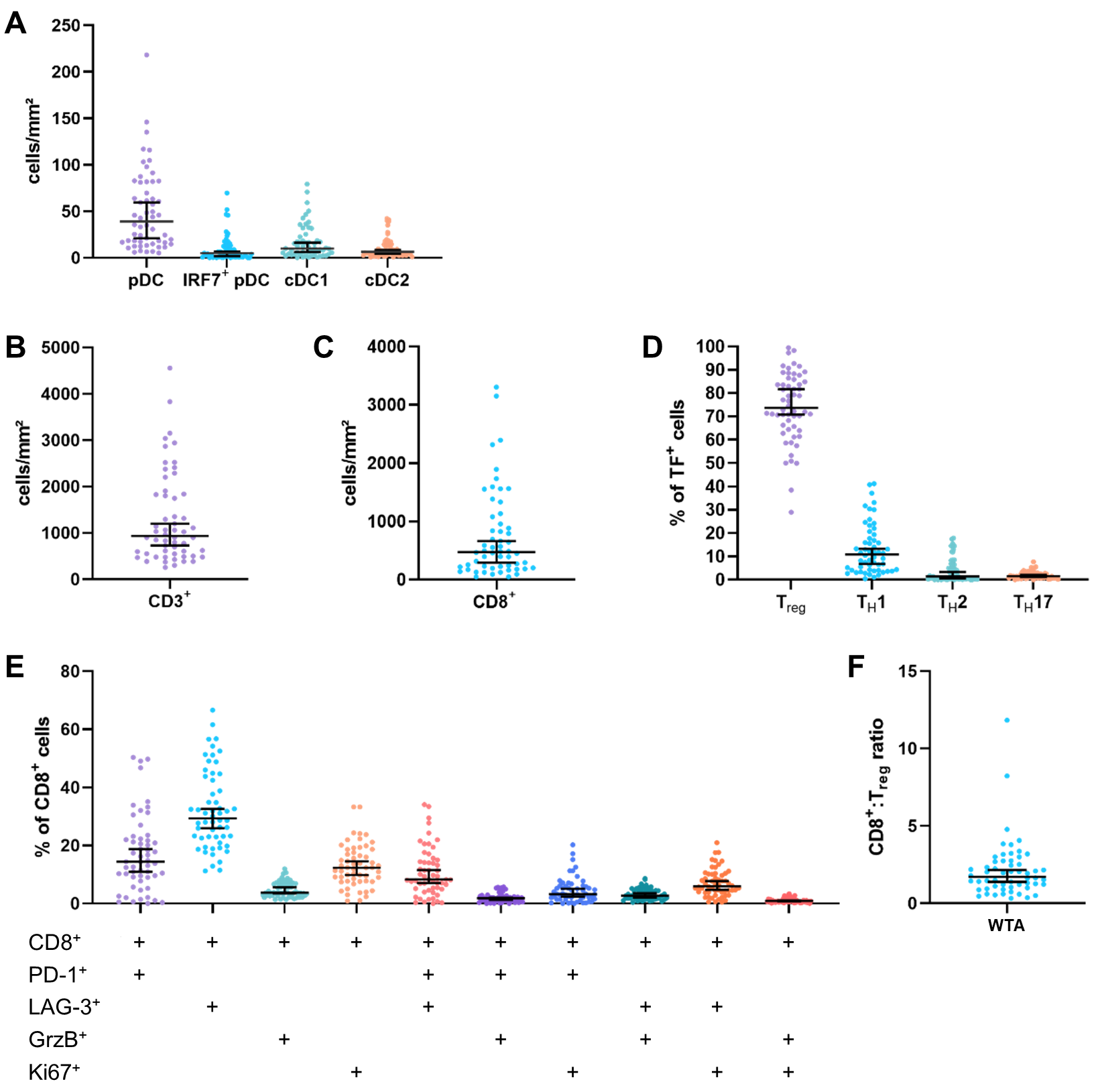


**Supplementary Figure 3:** **Quantified immune cell infiltration of the whole tumor area (WTA) of the whole HNSCC cohort.** Frequency of **A)** DCs and **B)** CD3^+^ and **C)** CD8^+^ T cells. **D)** Proportion of T_H_ subpopulations on total T cells expressing any transcription factor (TF). **E)** Proportion of CD8^+^ subpopulations expression further functional markers on total CD8^+^ T cells. Median with 95% CI (confidence interval).


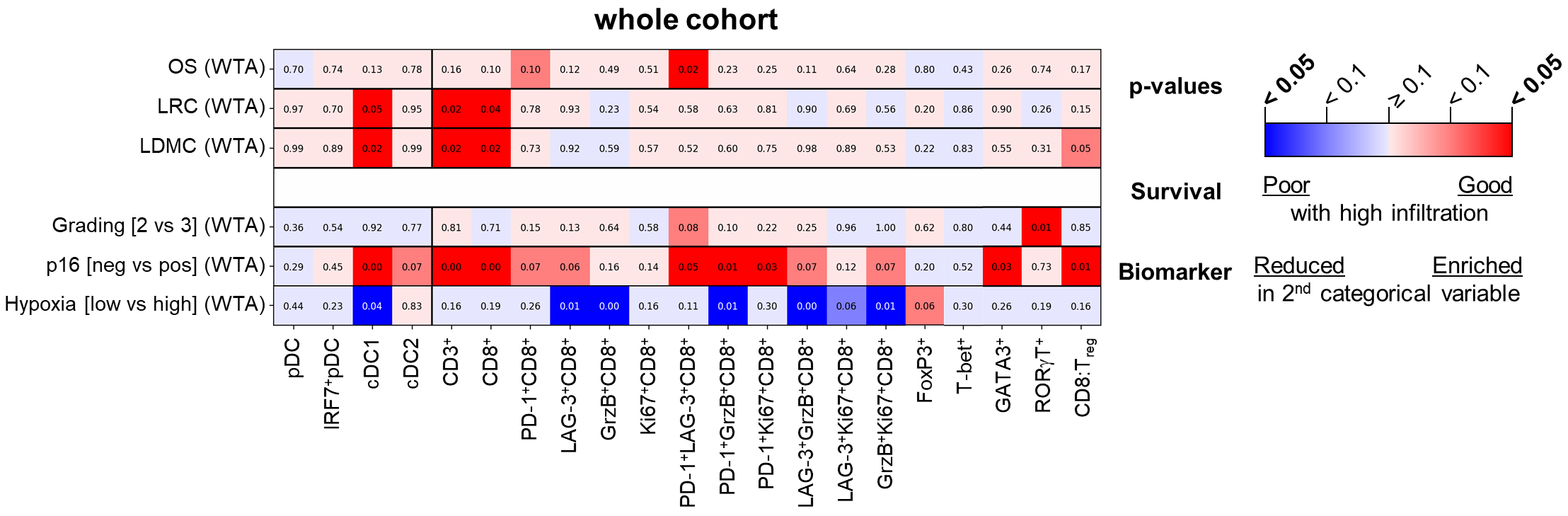


**Supplementary Figure 4:** **Quantified immune cell infiltration of the whole tumor area (WTA) with respect to survival and clinicopathological parameters of the whole HNSCC cohort.** Calculated p-values (survival: Log-rank test, clinical parameters: Mann-Whitney test) are depicted in the heatmap (red indicates i) a positive association of survival probabilities with tumor infiltration and ii) a higher immune cell frequencies in G3/p16+/Hypox15 high subcohorts; blue indicates i) a negative association of survival probabilities with tumor infiltration and ii) a higher immune cell frequencies in G2/p16-/Hypox15 low subcohorts).


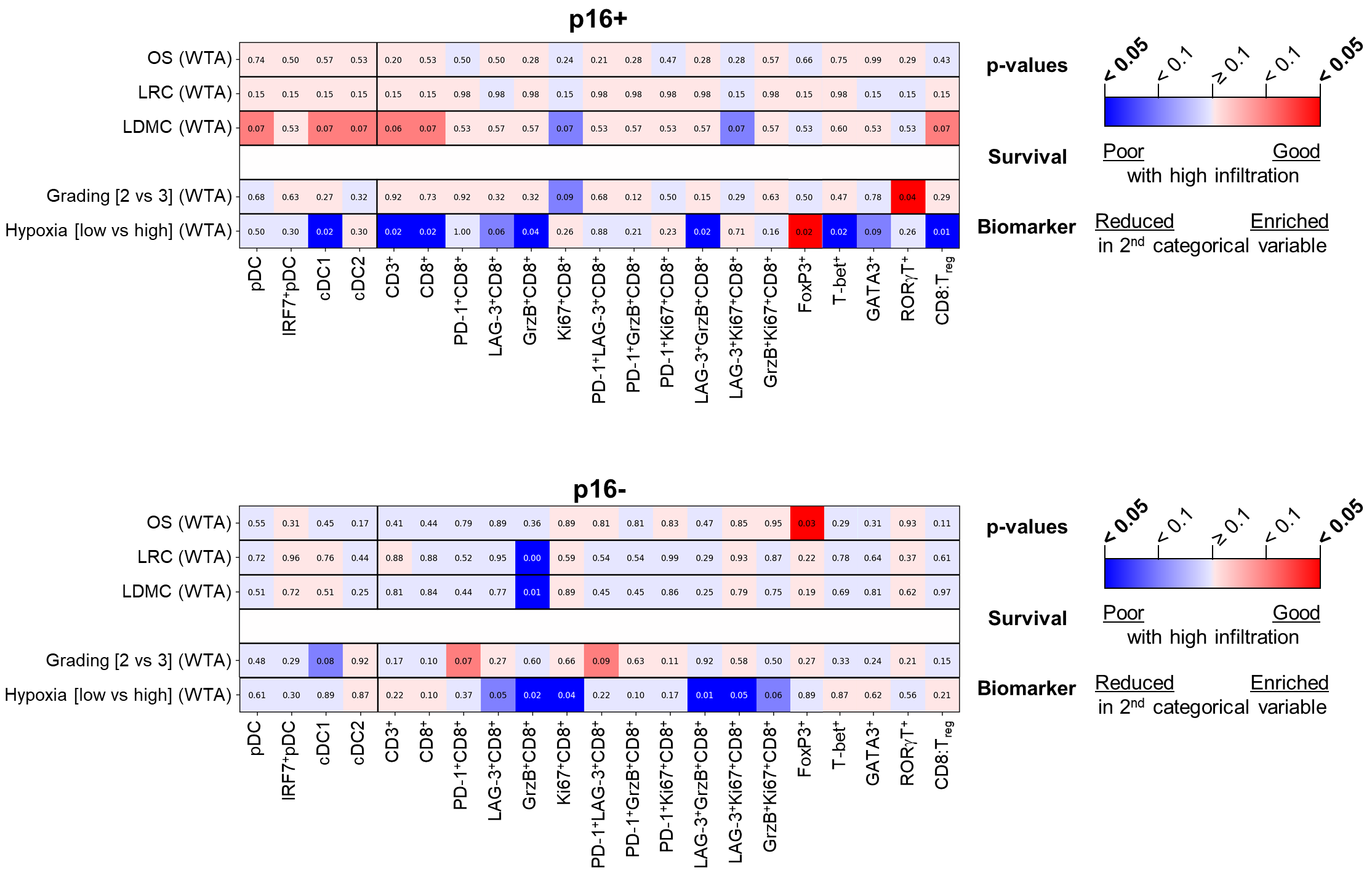


**Supplementary Figure 5:** **Quantified immune cell infiltration of the whole tumor area (WTA) with respect to survival and clinicopathological parameters of p16+ and p16- subcohorts.** Calculated p-values (survival: Log-rank test, clinical parameters: Mann-Whitney test) are depicted in the heatmap (red indicates i) a positive association of survival probabilities with tumor infiltration and ii) a higher immune cell frequencies in G3/Hypox15 high subcohorts; blue indicates i) a negative association of survival probabilities with tumor infiltration and ii) a higher immune cell frequencies in G2/Hypox15 low subcohorts).


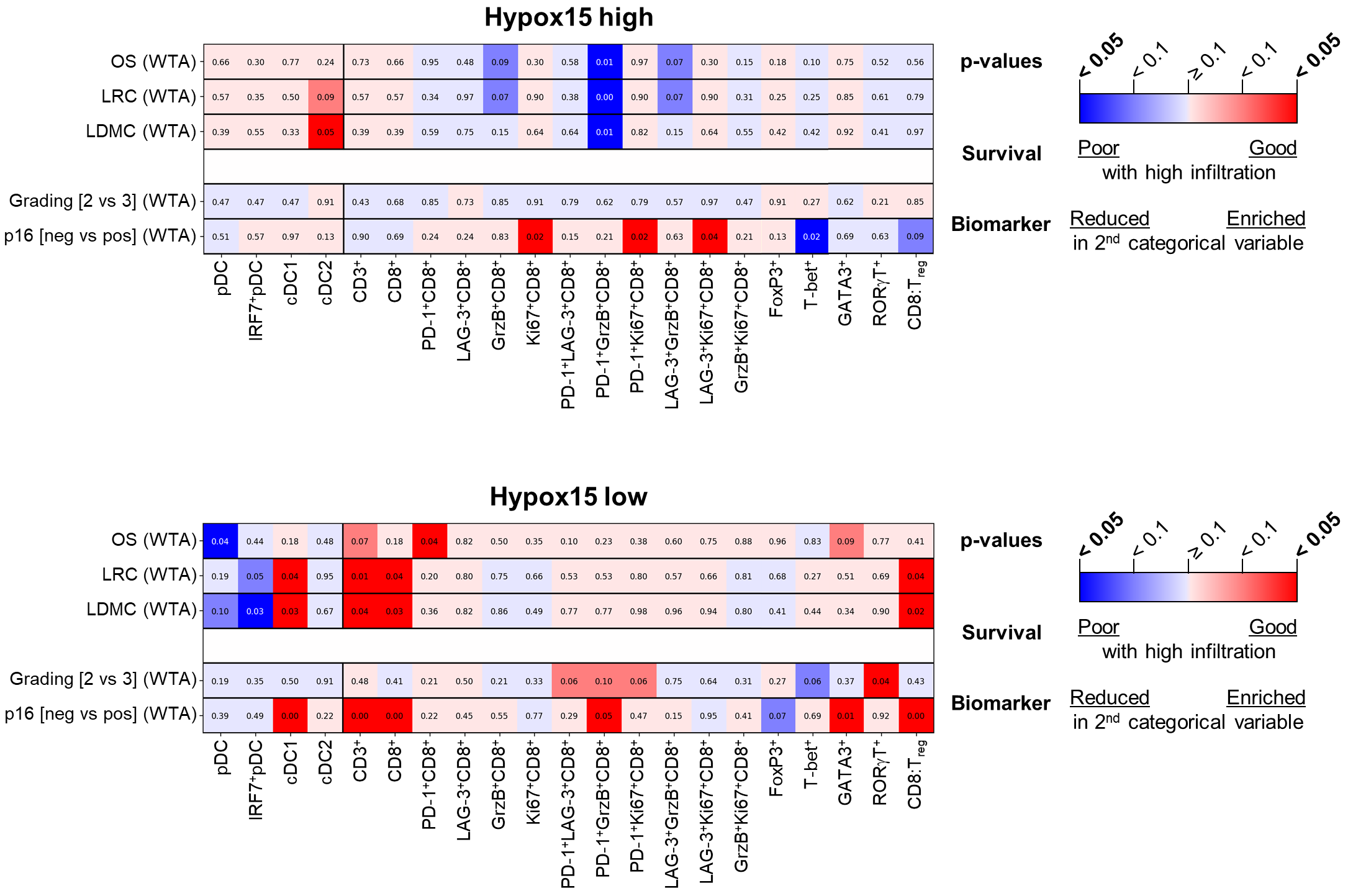


**Supplementary Figure 6:** **Quantified immune cell infiltration of the whole tumor area (WTA) with respect to survival and clinicopathological parameters of Hypox15 high and Hypox15 low subcohorts.** Calculated p-values (survival: Log-rank test, clinical parameters: Mann-Whitney test) are depicted in the heatmap (red indicates i) a positive association of survival probabilities with tumor infiltration and ii) a higher immune cell frequencies in G3/p16+ subcohorts; blue indicates i) a negative association of survival probabilities with tumor infiltration and ii) a higher immune cell frequencies in G2/p16- subcohorts).


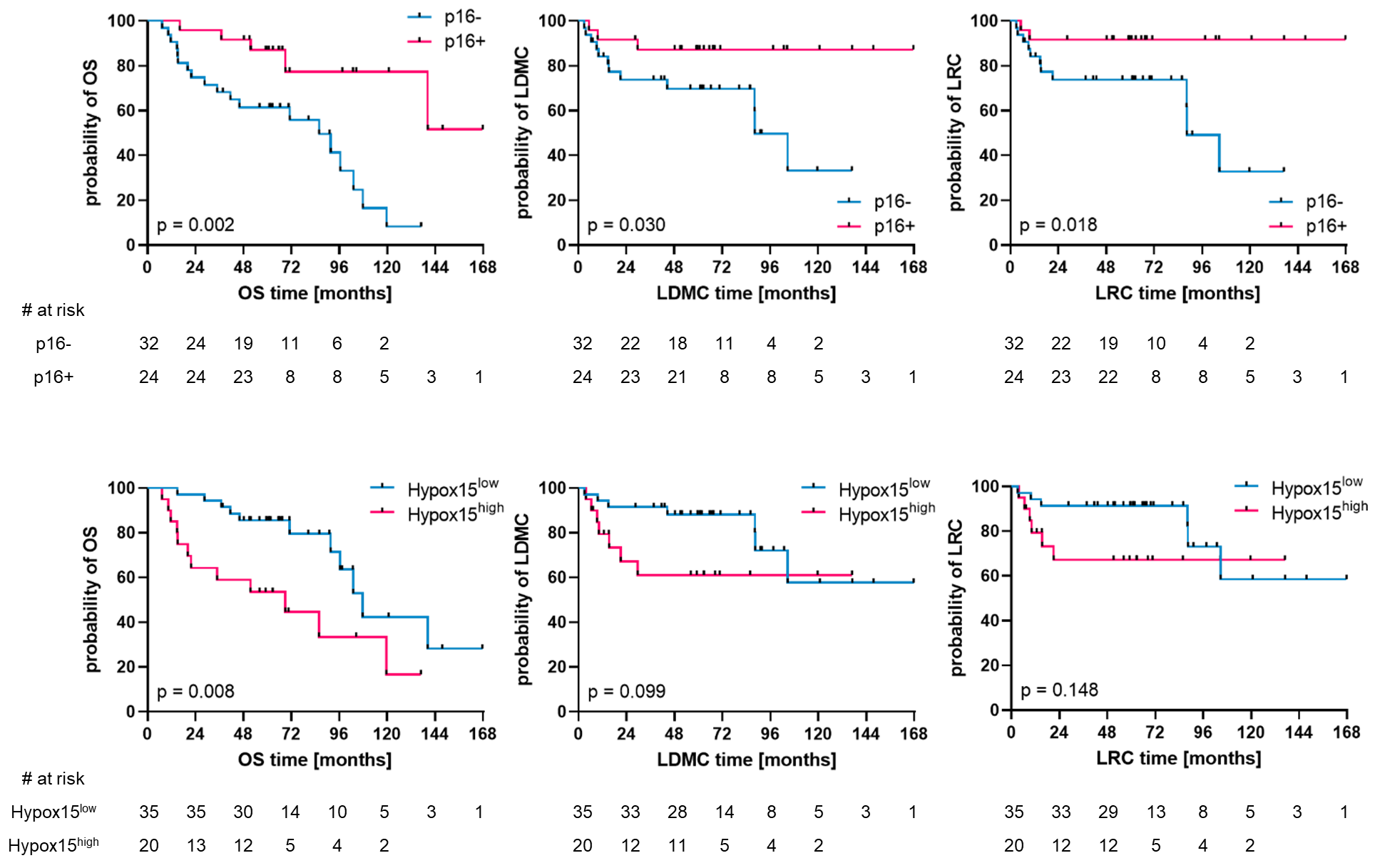


**Supplementary Figure 7: Clinical outcome of HNSCC subcohorts based on p16 positivity and hypoxia status.** Kaplan-Meier curves show patient outcome (OS, LRC, LDMC) for p16- or p16+ HNSCC patients (top) as well as for Hypox15^low^ or Hypox15^high^ HNSCC patients (bottom). p-values calculated by Log-rank test.


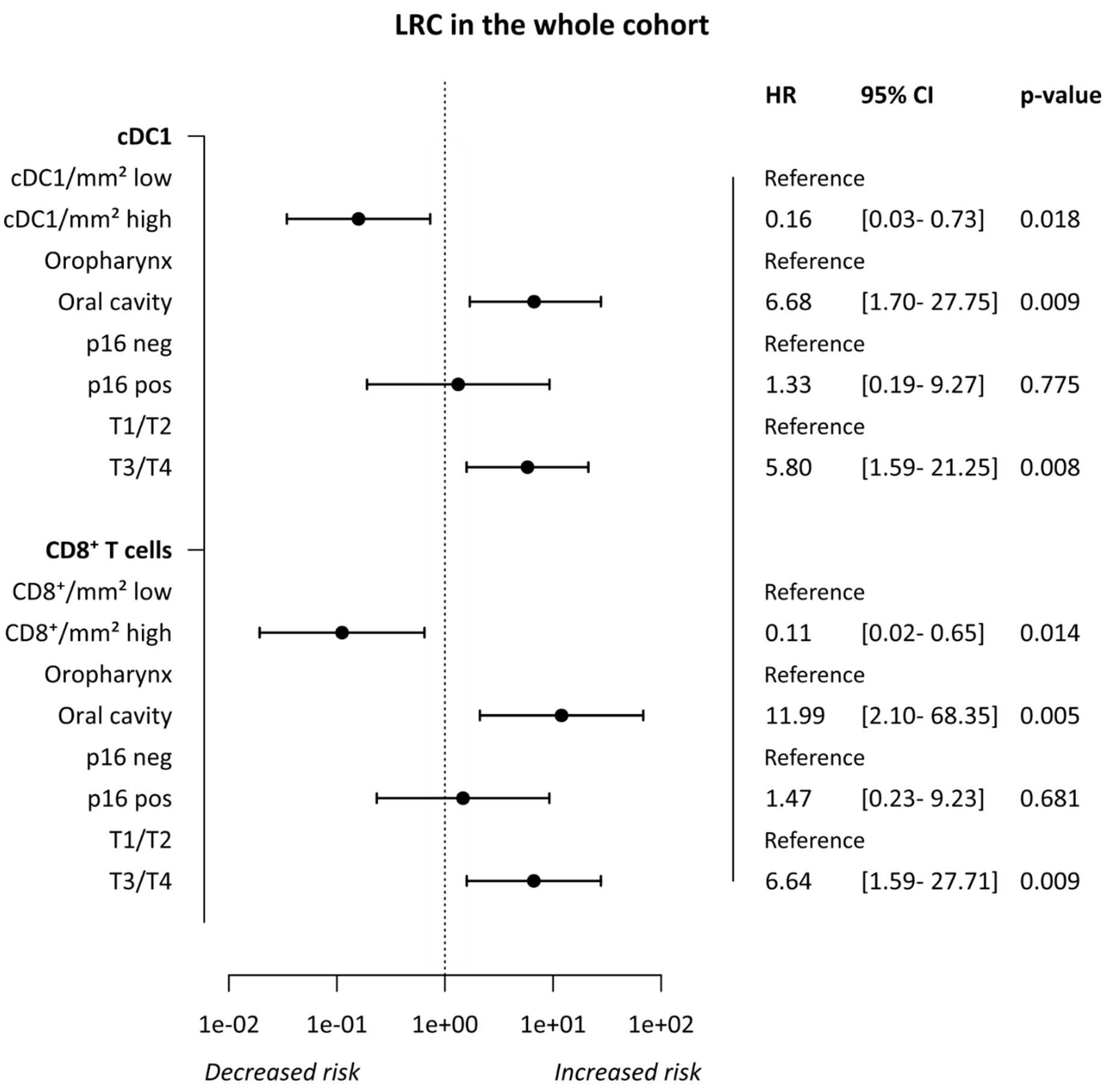


**Supplementary Figure 8: Multivariate Cox Regression for LRC in the whole HNSCC cohort.** Forest plots visualising Cox regression for cDC1 and CD8^+^ T-cells with respect to LRC and clinicopathological characteristics of the whole HNSCC cohort (localisation, p16 positivity, T stage).


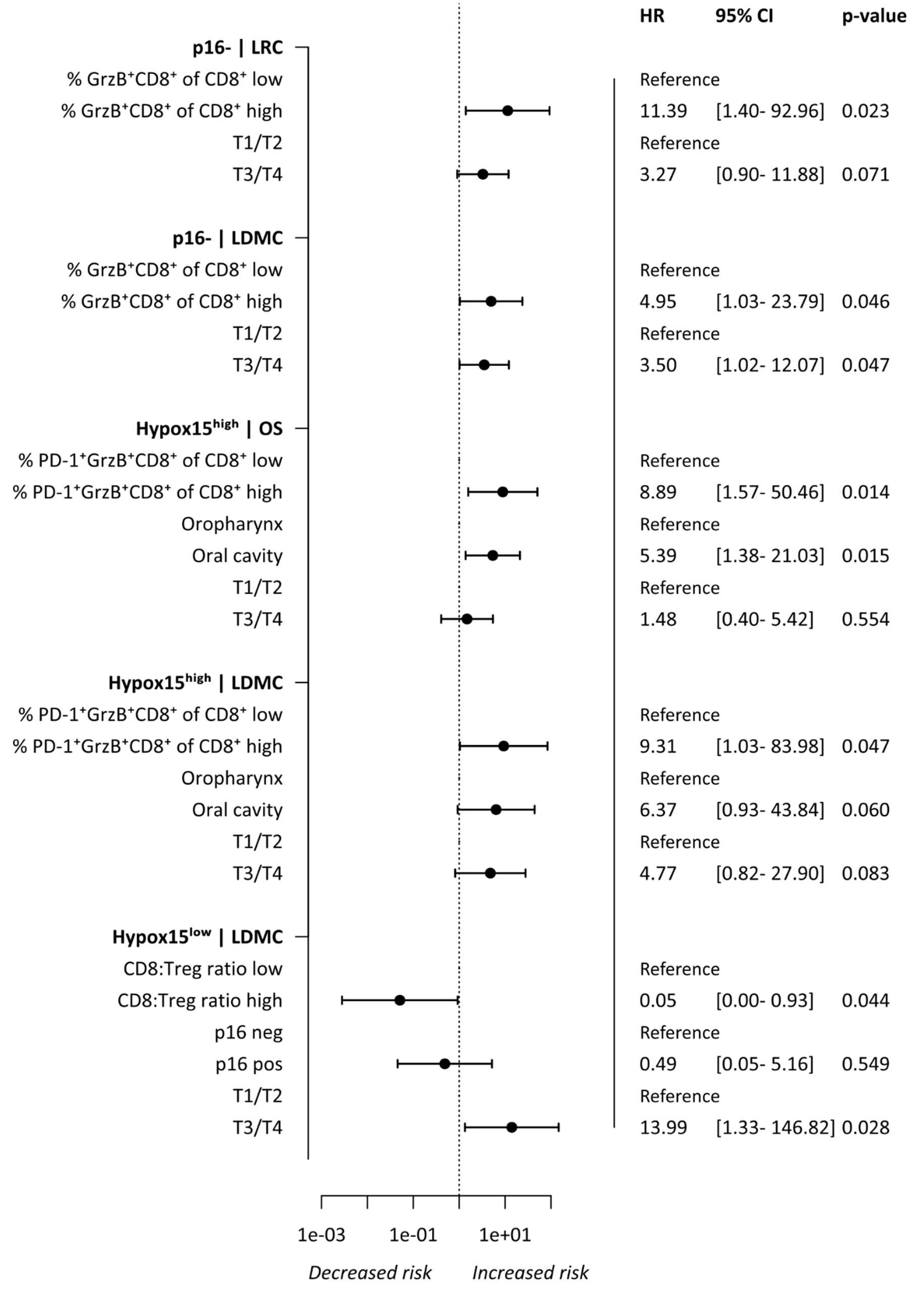


**Supplementary Figure 9: Multivariate Cox Regression for HNSCC subcohorts.** Forest plots visualising Cox regression for activated T-cells (GrzB^+^CD8^+^ and PD-1^+^GrzB^+^CD8^+^) and CD8:Treg ratio with respect to LRC, LDMC, or OS and clinicopathological characteristics of p16-, Hypox15^high^, and Hypox15^low^ subcohorts (localisation, p16 positivity, T stage).
